# Anthropogenic organic aerosol in Europe produced mainly through second-generation oxidation

**DOI:** 10.1038/s41561-025-01645-z

**Published:** 2025-03-10

**Authors:** Mao Xiao, Mingyi Wang, Bernhard Mentler, Olga Garmash, Houssni Lamkaddam, Ugo Molteni, Mario Simon, Lauri Ahonen, Antonio Amorim, Andrea Baccarini, Paulus Salomon Bauer, Dexian Chen, Randall Chiu, Lubna Dada, Jonathan Duplissy, Henning Finkenzeller, Lukas Fischer, Xu-Cheng He, Martin Heinritzi, Victoria Hofbauer, Changhyuk Kim, Andreas Kürten, Aleksandr Kvashnin, Katrianne Lehtipalo, Yuliang Liu, Huajun Mai, Vladimir Makhmutov, Serge Mathot, Roy Mauldin, Antti Onnela, Tuukka Petäjä, Lauriane L. J. Quéléver, Matti Rissanen, Simone Schuchmann, Mikko Sipilä, Dominik Stolzenburg, Yuri Stozhkov, Christian Tauber, António Tomé, Robert Wagner, Chao Yan, Boxing Yang, Penglin Ye, Qiaozi Zha, Joachim Curtius, Armin Hansel, Jasper Kirkby, Markku Kulmala, Rainer Volkamer, Paul M. Winkler, Douglas R. Worsnop, Wei Nie, Neil M. Donahue, Christopher R. Hoyle, Jianhui Jiang, Urs Baltensperger, Josef Dommen, Imad El Haddad

**Affiliations:** 1 PSI Center for Energy and Environmental Sciences, Paul Scherrer Institute, Villigen, Switzerland; 2 Center for Atmospheric Particle Studies, Carnegie Mellon University, Pittsburgh, PA USA; 3 Division of Chemistry and Chemical Engineering, California Institute of Technology, Pasadena, CA USA; 4 Institute of Ion Physics and Applied Physics, University of Innsbruck, Innsbruck, Austria; 5 Institute for Atmospheric and Earth System Research / Physics, University of Helsinki, Helsinki, Finland; 6 Department of Chemistry, University of Copenhagen, Copenhagen, Denmark; 7 WSL Swiss Federal Institute for Forest, Snow and Landscape Research, Birmensdorf, Switzerland; 8 Institute for Atmospheric and Environmental Sciences, Goethe University Frankfurt, Frankfurt am Main, Germany; 9 CENTRA and FCUL, University of Lisbon, Lisbon, Portugal; 10 School of Architecture, Civil and Environmental Engineering, École Polytechnique Fédérale de Lausanne, Lausanne, Switzerland; 11 Faculty of Physics, University of Vienna, Vienna, Austria; 12 Department of Chemistry and CIRES, University of Colorado, Boulder, CO USA; 13 Helsinki Institute of Physics, University of Helsinki, Helsinki, Finland; 14 School of Civil and Environmental Engineering, Pusan National University, Busan, Republic of Korea; 15 Lebedev Physical Institute, Russian Academy of Sciences, Moscow, Russian Federation; 16 Finnish Meteorological Institute, Helsinki, Finland; 17 Joint International Research Laboratory of Atmospheric and Earth System Sciences, School of Atmospheric Sciences, Nanjing University, Nanjing, China; 18 CERN, Geneva, Switzerland; 19 Department of Chemistry, Carnegie Mellon University, Pittsburgh, PA USA; 20 Department of Oceanic and Atmospheric Sciences, University of Colorado, Boulder, CO USA; 21 Aerosol Physics, Physics Unit, Engineering and Natural Sciences, Tampere University, Tampere, Finland; 22 Johannes Gutenberg University Mainz, Mainz, Germany; 23 Institute of Materials Chemistry, TU Wien, Vienna, Austria; 24 IDL-Universidade da Beira Interior, Rua Mateus D’Avila e Bolama, Covilhã, Portugal; 25 Aerodyne Research Inc., Billerica, MA USA; 26 Institute for Atmospheric and Climate Science, ETH Zurich, Zurich, Switzerland; 27 Global Institute for Urban and Regional Sustainability, School of Ecological and Environmental Sciences, East China Normal University, Shanghai, China; 28 Institute of Eco-Chongming, East China Normal University, Shanghai, China

**Keywords:** Atmospheric chemistry, Environmental impact

## Abstract

Exposure to anthropogenic atmospheric aerosol is a major health issue, causing several million deaths per year worldwide. The oxidation of aromatic hydrocarbons from traffic and wood combustion is an important anthropogenic source of low-volatility species in secondary organic aerosol, especially in heavily polluted environments. It is not yet established whether the formation of anthropogenic secondary organic aerosol involves mainly rapid autoxidation, slower sequential oxidation steps or a combination of the two. Here we reproduced a typical urban haze in the ‘Cosmics Leaving Outdoor Droplets’ chamber at the European Organization for Nuclear Research and observed the dynamics of aromatic oxidation products during secondary organic aerosol growth on a molecular level to determine mechanisms underlying their production and removal. We demonstrate that sequential oxidation is required for substantial secondary organic aerosol formation. Second-generation oxidation decreases the products’ saturation vapour pressure by several orders of magnitude and increases the aromatic secondary organic aerosol yields from a few percent to a few tens of percent at typical atmospheric concentrations. Through regional modelling, we show that more than 70% of the exposure to anthropogenic organic aerosol in Europe arises from second-generation oxidation.

## Abstract (web summary)

Second-generation oxidation boosts the production of anthropogenic organic aerosol, according to an analysis of urban haze in an experimental chamber.

## Main

Atmospheric aerosol particles have major effects on Earth’s climate^1^ and human health^2^. Anthropogenic secondary organic aerosol (SOA), formed through the oxidation of gaseous precursors, is a key driver of haze events in urban settings^3,4^. This aerosol fraction has a high oxidative potential affecting human health^5^ and substantially contributes to air pollution-related mortality in cities on a global scale^6^. Aromatic hydrocarbons, emitted from combustion processes such as gasoline exhaust^7,8^ and residential wood combustion^9^, are recognized among the most efficient anthropogenic SOA precursors^10,11^. Their oxidation products dominate SOA growth during haze formation in several Chinese megacities^4,12^.

SOA mass yields reported for aromatics on the basis of chamber experiments span more than one order of magnitude^9^. This variability may reflect a real dependence of the volatility of aromatic oxidation products on the oxidation conditions, including NO_*x*_ levels^13,14^, or instead relate to experimental limitations, including vapour losses to chamber walls^15^. Aromatics react exclusively with OH radicals, forming hydroxycyclohexadienyl radicals, which further react with O_2_ to form phenolic compounds or peroxy radicals (RO_2_)^16^. Before termination with NO, HO_2_ or RO_2_ radicals, RO_2_ may undergo intramolecular hydrogen shifts, followed by the addition of molecular oxygen^17^. This process, termed autoxidation, rapidly produces highly oxygenated organic molecules (HOMs)^18–20^, contributing to SOA formation^21,22^. However, with SOA yields of ~10%, most of the oxidation products remain in the gas phase, and their subsequent second-generation oxidation may also contribute to HOM formation in the atmosphere^20,21^. Conversely, in chambers, these first-generation products can be readily lost to walls, resulting in a substantial underestimation of the role of second-generation chemistry in HOM and SOA formation. More than for other precursor types, for aromatics the OH reactivity of the oxidation products can increase strongly after the first generation. HOM yields have been found to increase with OH concentrations, indicating the importance of multi-generation oxidation for HOM formation^20,23^. As a sequence of OH-oxidation steps occurs simultaneously and cannot be experimentally separated, HOM yields from different generations of oxidation have never been determined. Hence, it is not yet known whether the formation of low-volatility species in aromatic SOA is driven mainly by rapid autoxidation during one oxidation step or instead by second- (or more) generation oxidation. Determining the dominant pathway is vitally important for quantifying the burden of aromatic SOA, its spatial and temporal variability in urban atmospheres and its health impact.

In this Article, we provide the experimental observations needed to understand anthropogenic SOA formation mechanistically and quantitatively in the atmosphere. We investigated the oxidation of three aromatic model compounds—toluene, 1,2,4-trimethylbenzene and naphthalene—at the ‘Cosmics Leaving Outdoor Droplets’ (CLOUD)^24^ facility at CERN (the European Organization for Nuclear Research) at 20 °C and 60% relative humidity. We identified the oxidation products contributing to SOA growth on a molecular level and determined their production and loss mechanisms on the basis of their dynamics in the chamber. We grouped these products into first- and second-generation oxidation products and estimated their formation yields. By implementing those yields in a regional air quality model, we quantified the impact of second-generation chemistry on the total anthropogenic organic aerosol (AOA) across Europe.

## Observation of HOMs from aromatic hydrocarbons

**Fig. 1:**
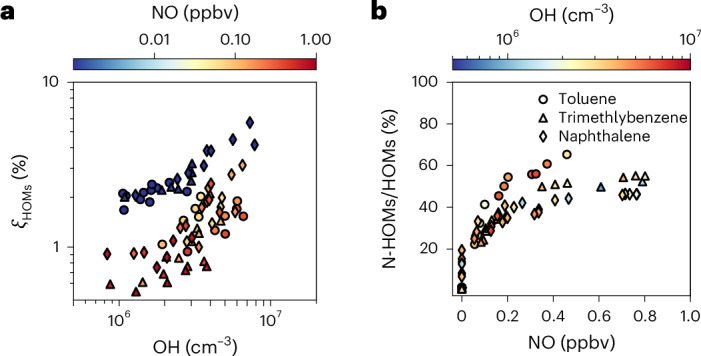
Apparent HOM yields and fraction of nitrogen-containing HOMs. **a**, *ξ*_HOMs_ from different precursors as a function of OH concentration. **b**, Ratio of nitrogen-containing HOMs (N-HOMs) to total HOMs as a function of NO. Toluene, 1,2,4-trimethylbenzene and naphthalene are marked by circles, triangles and diamonds, respectively. The colours of the symbols present average NO and OH concentrations in **a** and **b**, respectively. Yield data are from different VOC and NO_*x*_ injections as well as different light settings. which produced different OH levels (Supplementary Table 13).

**Extended Data Fig. 1:**
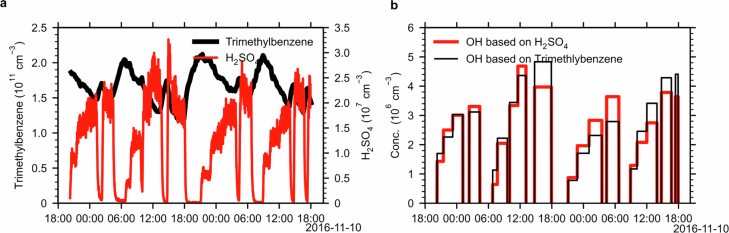
Comparison of OH concentration estimated based on H_2_SO_4_ and trimethylbenzene. (**a**) H_2_SO_4_ (red) and 1,2,4-trimethylbenzene (black) in trimethylbenzene oxidation experiments. (**b**) OH concentration estimated based on H_2_SO_4_ (red) and trimethylbenzene (black).

We observed HOM production for all precursors, shown in Fig. 1 as apparent HOMs mass yield, *ξ*_HOMs_. This is the ratio of the production rate of HOMs, *P*_HOM_, to the oxidation rate of the precursor aromatic compounds, *R*_ARO_ (Methods). OH concentrations are calculated on the basis of the decay of the organic precursors or the production of sulfuric acid (Extended Data Fig. 1). While *ξ*_HOMs_ shows moderate sensitivity to the precursor structure, it strongly depends on the OH and NO concentrations (Fig. 1a).

When the OH concentration increases from 1 × 10^6^ to 8 × 10^6^ cm^–3^, *ξ*_HOMs_ increases from 1.5% to 6.0% without NO and from 0.5% to 3.0% with NO. This suggests that multiple-generation reactions dominate the production of HOMs in aromatic systems, consistent with previous reports^20^. However, the 4% increase in the molar fraction of HOMs per 10^7^ cm^–3^ OH under NO_*x*_-free conditions at the CLOUD chamber is much higher than the increase reported for benzene HOMs of 0.25% per 10^7^ cm^–3^ OH from the Jülich plant atmosphere chamber^20^. This difference is due to the considerably higher vapour wall-loss rate in the Jülich chamber (1.1 × 10^–2^ s^–1^) compared with the CLOUD chamber (1.4 × 10^–3^ s^–1^). As we shall show in the following, vapour wall losses play a key role in the loss of second-generation products and their precursors from first-generation oxidation.

The NO reacts with RO_2_ to produce nitrate HOMs (NO + RO_2_→RONO_2_) or alkoxy radicals (NO + RO_2_→NO_2_ + RO), suppressing RO_2_ autoxidation. At high OH concentrations, the addition of NO reduces *ξ*_HOMs_ by a factor of 2, indicating that RO_2_ autoxidation remains important for producing HOMs even when most of the HOMs are produced through second-generation chemistry. By contrast, the yield of biogenic HOMs, which are produced mainly through autoxidation, is far more sensitive to NO_*x*_ concentrations^25^. In the CLOUD chamber, reactions with NO become the major sink of RO_2_ at NO concentrations far below urban levels (>0.2 ppbv), where the ratio of nitrogen-containing HOMs to total HOMs reaches a plateau of 50% (Fig. 1b and Extended Data Table 1).

## Identification of first- and second-generation products

**Fig. 2:**
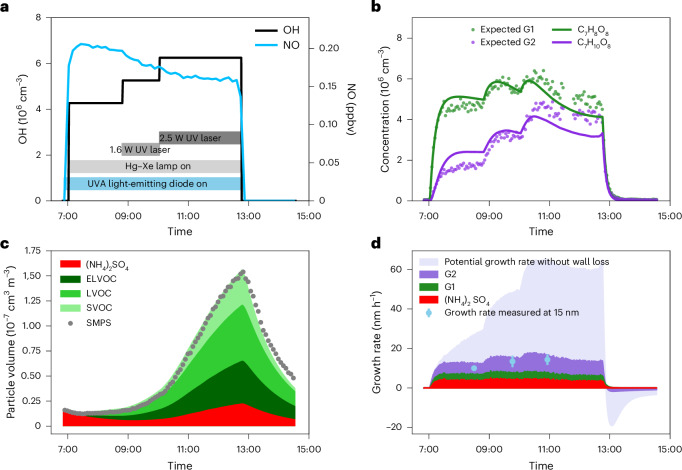
An example experiment of OH-initiated toluene oxidation in the presence of NO. **a**, OH (black line, left axis) and NO (blue line, right axis) concentrations of the experiment on 8 November 2016. **b**, Measured time series of C_7_H_8_O_8_ (green dots) and C_7_H_10_O_8_ (purple dots). Lines represent modelled time evolutions of first-generation (green) and second-generation (purple) oxidation products, assuming a wall-loss lifetime of 0.0014 s^–1^ for both products. **c**, Secondary aerosol mass closure in the CLOUD chamber. The coloured areas are modelled particle volumes based on gas-phase measurements using PTR3 and NO_3_-CIMS data. The red, dark green and light green areas are the modelled volume concentrations of sulfate, ELVOCs and LVOCs, respectively. The solid circles represent the particle volume based on the scanning mobility particle sizer (SMPS) measurements, highlighting the great model–measurement agreement. **d**, Growth rate contributions of (NH_4_)_2_SO_4_, first- and second-generation oxidation products for 15 nm particles. Sky blue circles show measured growth rates for 10 to 20 nm particles ± 95% fitting confidence interval using the appearance time method. Lavender area is potential growth rate contribution of SOA if first-generation oxidation products are not lost to the chamber walls (Supplementary Figs. 4 and 5).

Figure 2 shows an example experiment exploring toluene oxidation in the presence of NO_*x*_, SO_2_ and NH_3_. The OH concentration was ramped up by increasing the UV radiation, leading to a decrease in NO concentrations (Fig. 2a).

We examined the appearance times of the oxidation products and their time evolution at different OH concentrations to identify the mechanism of their production, their loss and the loss of their precursors. Second-generation products are characterized by a delayed appearance time and a notable increase in their concentration with increasing OH compared with their first-generation precursors. The product appearance times further help to constrain their production mechanisms in the gas phase. Their delayed appearance time separates them from first-generation products formed through the HO_2_ + RO_2_ or RO_2_ + RO_2_ pathways, whose apparent yield may also correlate with the OH concentration due to the covariance of OH with RO_2_ and HO_2_ (Supplementary Fig. 1).

**Extended Data Fig. 2:**
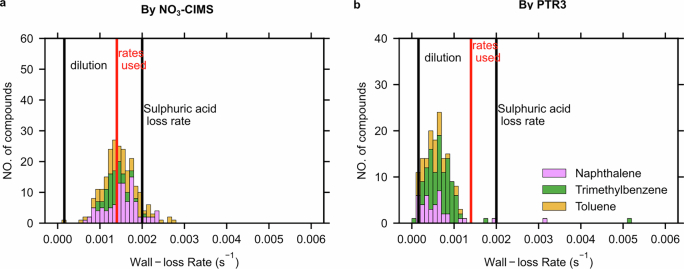
Wall-loss rates of oxidation products measured by (a) NO_3_-CIMS and (b) PTR3. The NO_3_-CIMS detects mostly HOMs (LVOCs and ELVOCs) which are irreversibly lost to the wall while the PTR3 detects mostly SVOCs which are not or with a low sticking coefficient lost to the wall. For sulfuric acid (98 Da) a wall loss rate of 0.002 s^−1^ was measured. Assuming an average molecular mass of 200 Da for HOMs and considering a mass^1/2^ dependence for the diffusivity, the average wall loss rate for HOMs is expected to be around 0.0014 s^−1^, shown as a red line in the figures. This rate is in good agreement with the wall loss rates of HOMs measured with the NO_3_-CIMS (0.00146 s^−1^, with a 10^th^ percentile of 0.00099 s^−1^ and a 90^th^ percentile of 0.00197 s^−1^).

In a continuously stirred reactor with steady reactant flows in and out, species will be lost principally via either dilution or wall deposition. The dilution timescale was 1.7 h. We measured a wall-loss lifetime of ~12 min (Extended Data Fig. 2) in the absence of production when the ultraviolet (UV) lights were turned off. We modelled the time evolution of first-generation (G1) and second-generation (G2) products with lifetimes controlled by dilution or wall loss. Case G1_d_ is dilution controlled and G1_w_ is wall-loss controlled. For products from G1_d_, G2_dd_ is dilution controlled and G2_dw_ is wall-loss controlled. For products from G1_w_, G2_ww_ is wall-loss controlled. We determine which case applies to each individual species on the basis of their time evolution.

**Extended Data Fig. 3:**
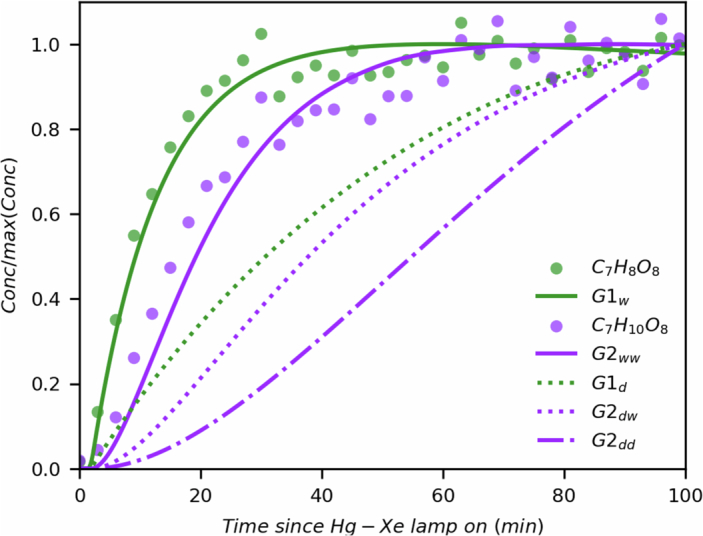
Appearance time of oxidation products after UV lights turned on. Markers are C_7_H_8_O_8_ (green) and C_7_H_10_O_8_ (purple) measured by NO_3_-CIMS. The green solid line (G1_w_) represents modelled OH initiated toluene oxidation products using the measured wall-loss lifetime of C_7_H_8_O_8_. The green dotted line (G1_d_) represents modelled OH-initiated toluene oxidation products assuming only dilution loss. The solid purple (G2_ww_) line represents the modelled time evolution of a second-generation product with wall-loss lifetime produced from the oxidation of a first-generation oxidation product that is also lost to chamber walls. Dotted (G2_dw_) and dotted-dashed (G2_dd_) purple lines represent second-generation products lost to the wall and by dilution, respectively, from a first-generation product with dilution lifetime. The figure highlights quite readily the differences in the dynamics of oxidation products depending on their formation and sink mechanisms.

**Extended Data Fig. 4:**
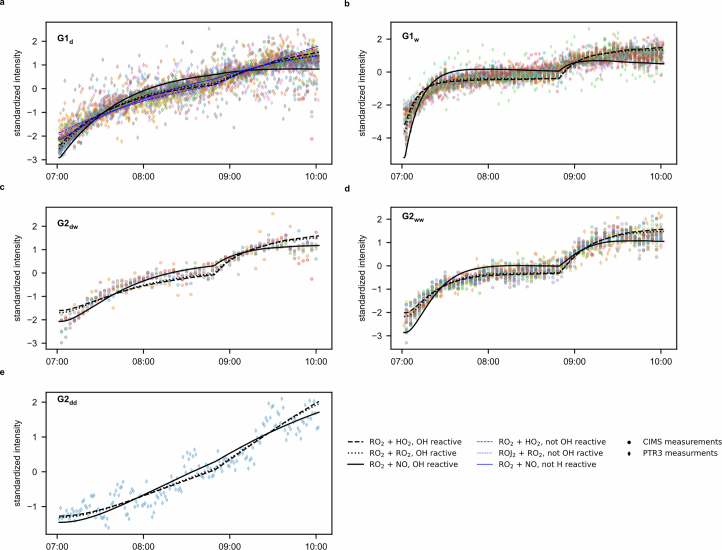
Generalized analysis of the temporal behavior of the toluene oxidation products in the CLOUD chamber. Circles and diamonds are compounds detected by NO_3_-CIMS or PTR-MS, respectively. **a**, G1_d_: First-generation products with dilution lifetime control. Black lines are modelled OH-reactive (*k*_OH_ = 4.65×10^−11 ^cm^3 ^s^−1^) G1_d_ produced by RO_2_ + NO reaction (solid line), RO_2_ + HO_2_ (dotted line) or RO_2_ + R’O_2_ (dashed line). Blue lines are the same as the black lines but assuming non-additional reactions with OH. **b**, G1_w_: First-generation products with wall-loss lifetime control. Lines are modelled G1_w_ produced by RO_2_ + NO reaction (solid line), RO_2_ + HO_2_ (dashed line) or RO_2_ + R’O_2_ (dotted line). **c**, G2_dw_: Second-generation oxidation products from G1_d_ with wall-loss lifetime control. Lines are modelled G2_dw_ considering different RO_2_ sinks. **d**, G2_ww_: Second-generation oxidation products from G1_w_ with wall-loss lifetime control. Lines are modelled G2_ww_ considering different RO_2_ sinks. **e**, G2_dd_: Second-generation oxidation products from G1d with dilution loss control. Black lines are modelled G2_dd_ considering different RO_2_ sinks.

Figure 2b shows the dynamics of two prominent toluene oxidation products, C_7_H_8_O_8_ and C_7_H_10_O_8_, at different OH concentrations. In Extended Data Fig. 3, we plot the time series of the same products during the first 100 min after the UV light was turned on to highlight the difference in their appearance times. The evolution of C_7_H_8_O_8_ is consistent with the time response of first-generation products, G1_w_, with irreversible chamber wall loss. The stepwise increase of OH is followed by a small enhancement in C_7_H_8_O_8_. By contrast, C_7_H_10_O_8_ substantially increases with increasing OH, consistent with the expected time behaviour of second-generation products, G2_ww_, formed from the oxidation of first-generation products having a wall-loss-defined lifetime. Although both C_7_H_8_O_8_ and C_7_H_10_O_8_ appear to have an appreciable wall loss, only the C_7_H_8_O_8_ appearance is consistent with that of first-generation oxidation products, while C_7_H_10_O_8_ shows a delayed appearance (Extended Data Fig. 3). The analysis in Fig. 2b and Extended Data Fig. 3 demonstrates that the appearance times of the oxidation products and their overall time evolution with varying OH concentration constrain their production through first- versus second-generation oxidation as well as their loss mechanism. We generalize this analysis to all oxidation products detected by nitrate ion-based chemical ionization mass spectrometry (NO_3_-CIMS) and proton transfer time-of-flight mass spectrometry (PTR3; Extended Data Fig. 4) and calculate their wall-loss-corrected yields.

**Fig. 3:**
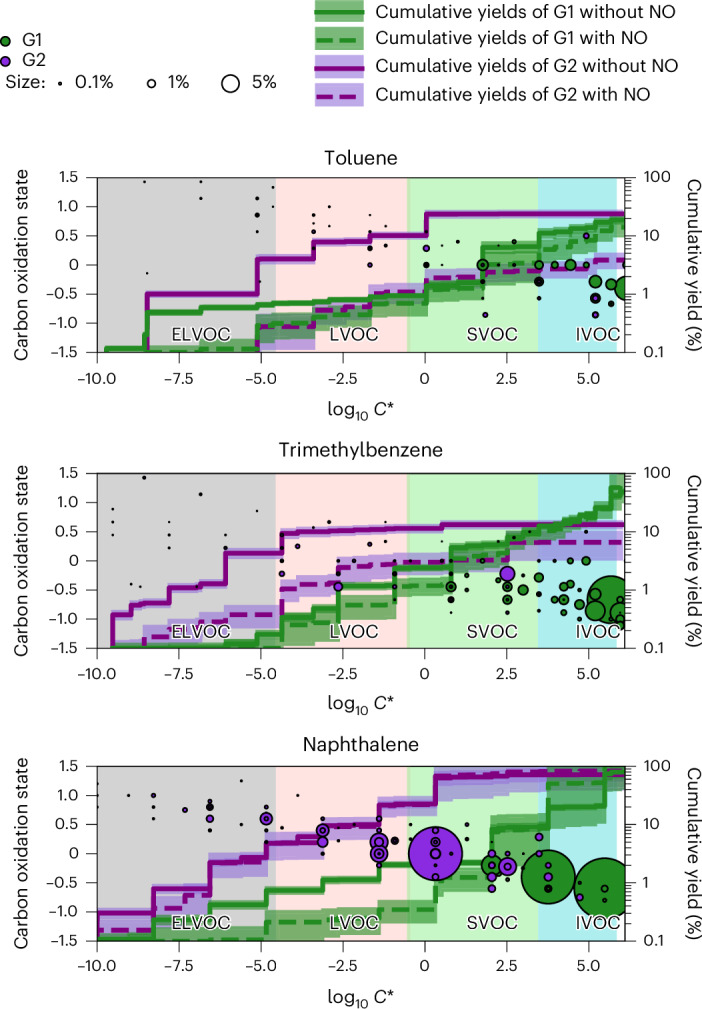
2D VBS of oxidation products of toluene, trimethylbenzene and naphthalene. The background colours indicate the C* ranges of ELVOCs (grey), LVOCs (pink), SVOCs (green) and IVOCs (blue). G1 (green) and G2 (purple) oxidation products are plotted as carbon oxidation states (${\mathrm{O}}{\overline{{\mathrm{S}}_{\mathrm{c}}}}$) versus log_10_ C* with marker areas proportional to their mass yields with NO. Coloured lines show the mean cumulative mass yields (right) of G1 (green) and G2 (purple) products with NO (dashed) or without NO (solid). Shaded areas show standard deviations of different chamber fillings or light conditions. Yields with NO are determined from experiments with NO concentration higher than 0.2 ppbv. OH concentrations are in the range of 0.8–8.7 × 10^6^ cm^−3^. Supplementary Tables 1–12 present the yields of individual compounds, associated standard deviations based on experimental variability, volatility and assignment to G1 versus G2 products.

**Extended Data Fig. 5:**
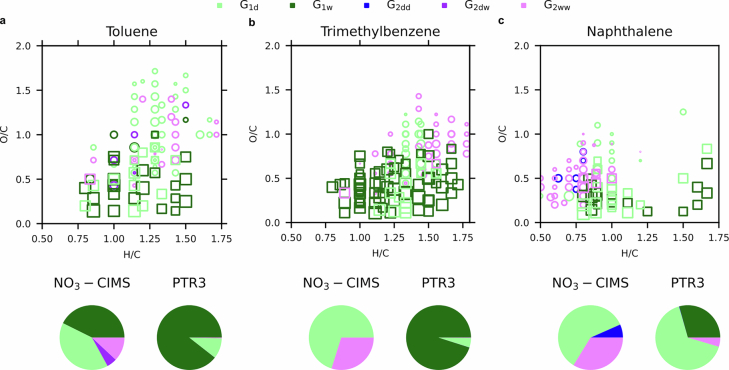
Van Krevelen diagrams and mass fractions of (a) toluene, (b) trimethylbenzene and (c) naphthalene oxidation products measured by NO_3_-CIMS and PTR3. Circles are oxidation products detected by NO_3_-CIMS; squares are oxidation products detected by PTR-MS. First-generation products with dilution lifetime (G1_d_) are colored in mint green, first-generation products with wall-loss lifetime (G1_w_) are in forest green, second-generation products from G1_d_ with dilution lifetime (G2_dd_) are in blue, second-generation products from G1_d_ with wall-loss lifetime (G2_dw_) are in lavender and second-generation products from G1_w_ with wall-loss lifetime (G2_ww_) are in pink. The areas of the symbols are proportional to the logarithm of the concentration of the compounds. Molecules detected by PTR3 with less than 4 carbon atoms are not shown. Fractions of the compounds detected by PTR-MS and NO_3_-CIMS are shown in the pie charts. Mass fractions measured by NO_3_-CIMS and PTR-MS cannot be compared since both instruments detect different ranges of oxygenated compounds.

In Fig. 3, we display the detected and categorized oxidation products of aromatic precursors in the presence of NO_*x*_ in a two-dimensional (2D) space with volatility on the *x* axis and carbon oxidation state (${\mathrm{O}}{\overline{{\mathrm{S}}_{\mathrm{c}}}}$) on the *y* axis (2D volatility basis set (VBS)). We measure and parameterize the volatility in terms of saturation vapour concentrations (C*, in µg m^–3^) using calibrated thermal desorption profiles^21^. The oxidation products span a wide range of C* with ${\mathrm{O}}{\overline{{\mathrm{S}}_{\mathrm{c}}}}$ decreasing from low to high volatilities. The second-generation products are characterized by higher ${\mathrm{O}}{\overline{{\mathrm{S}}_{\mathrm{c}}}}$ and several orders of magnitude lower C* compared with the first-generation products. The product distributions detected by PTR3 and NO_3_-CIMS are displayed as pie charts in Extended Data Fig. 5. The overwhelming majority of the species detected by PTR3 are first-generation products and more volatile (O/C ≲ 0.5), while almost all the low-volatility species (O/C ≳ 0.5) are detected by NO_3_-CIMS, and an important fraction of these are second-generation products. The NO_3_-CIMS detects mostly extremely low-volatility organic compounds (ELVOCs) and low-volatility organic compounds (LVOCs), which are irreversibly lost to the wall, while the PTR3 detects mostly semi-volatile organic compounds (SVOCs), which are not or reversibly lost to the wall.

Figure 3 and Extended Data Fig. 5 demonstrate that second-generation oxidation is necessary for a substantial production of low-volatility species in these aromatic systems. Most of the second-generation species and many of their precursors are lost relatively quickly to the chamber walls and therefore not accessible to most SOA yield analyses from smog chambers.

## Influence of second-generation oxidation on SOA yields

Aerosol growth in our experiments results from the condensation of sulfuric acid and the aromatic oxidation products. We modelled growth on the basis of the gas-phase concentrations of organic and inorganic condensable compounds measured by PTR3 and NO_3_-CIMS.

For toluene, we obtained an excellent agreement between measured and modelled particle volume (Fig. 2c). We also found similar agreement for trimethylbenzene and naphthalene (Supplementary Figs. 2 and 3), indicating that we accurately measured most condensable products and their saturation concentrations. Such agreement also suggests that aerosol growth from aromatic hydrocarbon oxidation can be explained by the condensation of condensable vapours produced in the gas phase, without invoking particle-phase processes, at least under our timescales and conditions. In a toluene oxidation experiment at 6 × 10^6^ OH and 180 pptv NO, second-generation products contribute 57% of the observed SOA mass (Fig. 2d).

The wall loss of the first-generation precursors largely prevents their aging into second-generation products with lower volatility. Unlike in the atmosphere, even SVOC species are efficiently lost to the walls instead of equilibrating with particles; therefore, in the atmosphere, second-generation chemistry is even more important than observed in the chamber. Removing the chamber wall losses from our model increases particle growth rates tenfold and the contribution of second-generation products to the total SOA from 57% to 89% (Fig. 2d). An important fraction of first-generation intermediate-volatility organic compounds (IVOCs) and SVOCs, which accounts for more than 95% of the molar yield after the first oxidation step, is converted into LVOCs and ELVOCs with a second oxidation step.

In Fig. 3, we present the wall-loss-corrected cumulative mass yields of oxidation products after one and two generations of oxidation for experiments with and without NO. At typical ambient organic aerosol (OA) concentrations, LVOCs and ELVOCs are always in the particle phase, IVOCs and VOCs are always in the gas phase, and SVOCs partition between the two phases. We note that the calculated yields (Fig. 3 and Supplementary Tables 1–13) depend on the assumed reaction-rate coefficient of first-generation products with OH (*k*_G1_ = 4.7 × 10^–11^ molecule^–1^ cm^3^ s^–1^). However, this dependence is weak compared with other factors driving the observed range of calculated yields. The calculated yields resulting from our analysis (Supplementary Table 13) and the assumed reaction-rate coefficient should not be used independently in models.

First-generation oxidation yields only 1–2% of LVOCs and ELVOCs (Fig. 3), while second-generation chemistry greatly increases their yields, by up to tenfold. The presence of NO lowers the formation of ELVOCS and LVOCS, especially for second-generation toluene and trimethylbenzene products. As product volatility drops by several orders of magnitude, aromatic SOA yields increase with aging. At typical atmospheric OA concentrations of 10 μg m^–3^ (log_10_C* = 1), over 90% of the first-generation products stay in the gas phase, reacting with OH to form less volatile second-generation products, increasing SOA yields by a few times (toluene) and up to more than tenfold (naphthalene). Thus, second-generation chemistry drives around 90% of aromatic HOM and SOA formation at atmospheric conditions.

In Methods, we quantify the errors associated with our yield analysis. We show that for lumped volatility bins, the error on the volatility (*x* axis on Fig. 3) is within a factor of 3, while the error on the yield of each volatility bin (secondary *y* axis on Fig. 3) is around a factor of 1.8. Therefore, errors in our analysis do not affect our conclusions given the magnitude of the effect of second-generation chemistry.

NO_*x*_ has an important role in aromatic SOA production. NO suppresses RO_2_ autoxidation during the first and second oxidation steps. It can compete with HO_2_/RO_2_ reacting with RO_2_, adding –NO_2_ groups instead of –OOH/–OH groups, reducing dimer formation rates and forming RO radicals that lead to the production of higher volatility carbonyl compounds. This causes lower SOA yields in our high NO_*x*_ experiments. Furthermore, at high NO_*x*_ conditions, the NO_3_ radical becomes a key oxidant, efficiently reacting with oxygenated aromatic products from first-generation chemistry and driving multiple-generation aging. We calculated up to 10^8^ cm^–3^ NO_3_ in some of our high NO_*x*_ experiments, which is still possible for a daytime atmosphere^26^ and high enough to compete with OH aging. At night, NO_3_ radicals are expected to be the major sink of first-generation products from aromatic oxidation.

For terpenes, mainly from biogenic emissions, rapid autoxidation during the first generation forms low-volatility products, immediately producing SOA. By contrast, aromatics require multiple-generation oxidation to form low-volatility species. Later-generation products have higher OH rate coefficients than their parent compounds^27^, promoting further oxidation and the addition of –OH groups, which more efficiently reduces volatility compared with –OOH groups from autoxidation. Thus, second-generation chemistry is crucial for producing low-volatility SOA from aromatics, and the higher SOA yields^28^ and reactivity of aromatic first-generation products make them highly efficient SOA precursors.

The importance of second-generation chemistry in the aromatic system means that the determination of SOA yields from single precursors and complex combustion emissions is especially sensitive to vapour wall-loss correction due to the wall loss of first-generation products. Previous studies have attempted to determine wall-loss-corrected SOA yields for different precursors^15^ and complex emissions^29^ but could not address the loss of vapours during multiple oxidation steps. Here, by following the dynamics of the SOA-forming oxidation products with direct observations, we effectively address the loss of these products and quantify SOA yields from different generations of oxidation.

## Impact of second-generation oxidation on OA pollution

To estimate the importance of first- versus second-generation chemistry for OA pollution in Europe, we modelled AOA from residential heating and fossil fuel burning using a VBS scheme based on the yields of first- and second-generation products determined from our experiments (Extended Data Table 2).

**Fig. 4:**
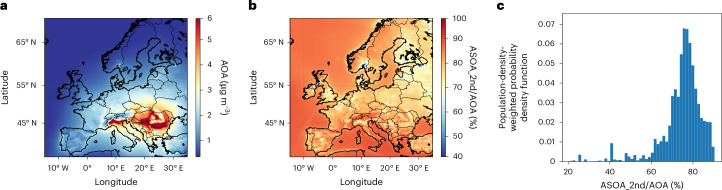
Annual AOA concentration and fraction of second-generation SOA. **a**, Spatial distribution of yearly averaged AOA. **b**, Fraction of second-generation SOA (ASOA_2nd) to AOA. **c**, The population-density-weighted probability density function (PDF) of ASOA_2nd to AOA.

**Extended Data Fig. 6:**
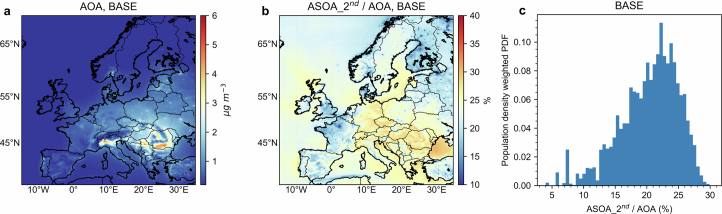
Annual AOA concentration and fraction of second-generation SOA using the default yields of CAMx. (**a**)Spatial distribution of yearly averaged anthropogenic OA (AOA), (**b**) fraction of the anthropogenic second-generation SOA to AOA, and (**c**) the population density weighted probability density function (PDF) of the ASOA_2^nd^/AOA values using the default yields of CAMx, for comparison with Fig. 4.

**Extended Data Fig. 7:**
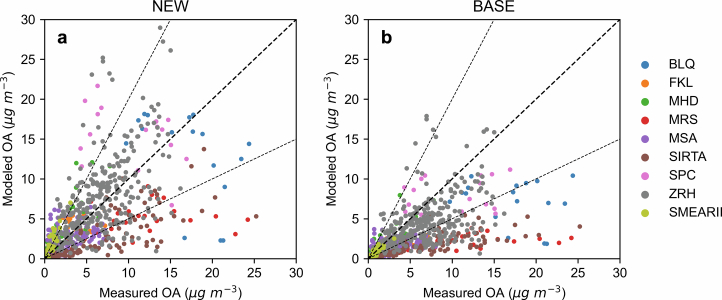
Comparison between the modeled and measured OA at nine ACSM/AMS stations over Europe using the (a) new and (b) base parameterizations. BLQ: Bologna, FKL: Finokalia, MHD: Mace Head, MRS: Marseille, MSA: Montsec, SIRTA: Paris Site Instrumental de Recherche par Télédétection Atmosphérique, SPC: San Pietro Capofiume, ZRH: Zurich, SMEARII: (Station for Measuring Forest Ecosystem–Aerosol Relations) II Hyytiälä. Detailed information and time span of each station can be found in Jiang et al.^44^.

Figure 4a shows annual AOA concentrations, while Extended Data Fig. 6 shows the same for a standard VBS scheme. Extended Data Fig. 7 compares these two model results with measurements at nine stations in Europe, indicating that modelled concentrations using either the base or new parameterizations exhibit moderate correlations with the observed daily concentrations. However, the model with updated parameters reproduces the observations much more accurately, with a reduction in the mean bias from –61% to –17% (Extended Data Table 3).

The highest concentration occurs in eastern Europe, with an annual average value of 6.0 µg m^–3^, mostly due to residential burning emissions. Weighted by population, second-generation chemistry contributes 71% of AOA across Europe. This is 3.5 times higher than previous assessments (Fig. 4 and Extended Data Fig. 6), underscoring the substantial limitations of previous chamber parameterizations in accurately constraining the production and loss mechanisms of aromatic products in absence of real-time molecular measurements. While primary emissions and first-generation products remain non-negligible in urban hotspots, second-generation products prevail in suburban and rural locations due to the time needed for their production.

For environments with high emissions of aromatic compounds, multiple-generation oxidation defines SOA spatial distribution and evaporation behaviour. It may explain the high concentrations of low-volatility oxygenated organic aerosols with an aerosol mass spectrometer relatively far from precursor emissions. Our results also imply that first-generation oxidation products of aromatic compounds and not direct aromatic emissions are the main anthropogenic SOA precursors in ambient environments.

**Fig. 5:**
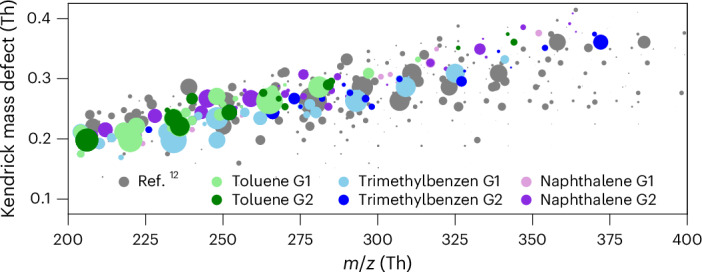
Comparison of oxidation products from chamber and ambient measurements. Kendrick mass defect of the aromatic oxidation products in ref. ^12^ compared with those detected in our chamber at high NO_*X*_ from the three precursors. Compounds in common with ref. ^12^ are coloured according to the precursor and first or second generation. Compounds detected in ref. ^12^ but not in our chamber are shown in grey.

Reference ^12^ demonstrated that aromatic oxidation products play a key role in SOA growth during haze events in Chinese megacities, with condensation fluxes far exceeding those from aliphatic compounds^12^. We provide the laboratory evidence demonstrating that a large portion of these products stems from second-generation chemistry. The direct comparisons between ambient observations (Fig. 2 in ref. ^12^) and our laboratory molecular measurements reveal a remarkable resemblance in the distribution of aromatic oxidation products (Fig. 5), with many compounds in common. These products have a carbon number ranging from 6 to 10, an O/C of ~0.5 and a double-bond equivalent between 3 and 5 (0.8 < H/C < 1.4).

Second-generation products constitute the largest fraction of anthropogenic OA pollution. Due to their high oxidation state, they may substantially influence the oxidative potential of OA and its associated health effects. This fraction is expected to dominate aerosol concentrations in populated areas, especially as SO_2_ and NO_*X*_ emissions decrease, highlighting the need for stricter regulations on aromatic emissions.

## Methods

### Experiments at the CLOUD facility

The experiments involving oxidation of aromatic precursors under urban conditions were conducted at the CLOUD facility^24^ at CERN during an intensive campaign in 2016 (CLOUD11). The 26.1 m^3^ electro-polished stainless-steel chamber was running in a flow-through mode with a continuous flow of 247 l min^–1^ synthetic air made from evaporation of cryogenic liquid nitrogen (Messer, 99.999%) and liquid oxygen (Messer, 99.999%) at a volume mixing ratio of 79:21 at 293 K and 60% relative humidity. The dilution lifetime is 1.6 h in the chamber.

Experiments with 1,2,4-trimethylbenzene (4–8 ppbv, Messer, 100 ppmv in N_2_), toluene (40 ppbv, Messer, 1,000 ppmv in N_2_) and naphthalene (0.1–10.0 ppbv, Sigma Aldrich, 99%) and SO_2_ (2 ppbv, CARBAGAS AG, 500 ppmv in N_2_) were performed in this study. When precursor gases reached the intended concentrations, experiments were started by turning on the UV light (a 4 W KrF excimer UV laser UVX at 248 nm and four 200 W Hamamatsu Hg–Xe lamps UVH at wavelengths between 250 and 450 nm) to produce OH from 40–100 ppbv ozone (O_3_) generated by exposing a small fraction of the air through a quartz tube surrounded by UVC lamps (wavelength < 240 nm). Experiments with NO_*x*_ were conducted by injecting NO_2_ in the chamber and using an LED sabre (LS3) at 385 nm to convert NO_2_ to NO. NO/NO_2_ in the experiments was around 0.05–0.10 (0.0025 s^–1^ maximum NO_2_ photolysis rate). To help nucleation and to have higher particle number concentrations, 1–2 ppbv NH_3_ (100 ppmv in N_2_, CARBAGAS AG) was injected. Experiments are stopped by turning off the UV source.

Gas monitors were used to measure O_3_ (Thermo Environmental Instruments TEI 49 C), SO_2_ (Thermo Fisher Scientific 42i-TLE) and NO (ECO Physics, CLD 780TR). NO_2_ was measured by a cavity attenuated phase shift NO_2_ monitor (Aerodyne Research) and a custom-made cavity enhanced differential optical absorption spectroscopy instrument. Chamber relative humidity was determined by dew-point mirrors (EdgeTech).

Concentrations of sulfuric acid and HOMs were measured with NO_3_-CIMS^30–32^. The fitting process of sulfuric acid and oxidation products employs a customized peak shape and a resolution function constructed on peaks dominated by a single composition (see Supplementary Fig. 6 for peak fitting). The fitting is performed sequentially from peaks of higher intensity towards lower intensity, with isotope peaks constrained by their abundance and composition with lower mass defects to higher mass defects allowing at most three nitrogen atoms in the formula. HOMs in this study are taken as the sum of organic molecules measured by the NO_3_-CIMS with six or more oxygen atoms and more than four carbon atoms. The toluene concentration was monitored by a quadrupole proton transfer reaction mass spectrometer (PTR-MS with quadrupole). For some experiments when the PTR-MS with quadrupole measurements were not available, we determined the toluene concentration from the injection rates (mass flow controller settings). A PTR3^33^ with a core sampling inlet transfers sample air through a tripole reaction chamber operated at 80 mbar and detects 1,2,4-trimethylbenzene, naphthalene and oxidation products from aromatic hydrocarbons down to 1 × 10^7^ cm^−3^. The PTR3 was calibrated with a gas standard containing 1 ppmv 3-hexanone, heptanone and *α*-pinene in nitrogen and a gas standard containing 1 ppmv naphthalene, trimethylbenzene and toluene, which was dynamically diluted by a factor of 1,000 in VOC-free air to reach 1 ppbv of each compound and at chamber humidity. The measurements of an iodide-adduct time-of-flight chemical ionization mass spectrometer equipped with a filter inlet for gases and aerosols (FIGAERO-ToF-CIMS) were presented by ref. ^21^.

The apparent HOMs yield (*ξ*_HOMs_) is determined assuming the oxidation products from aromatic precursors (OxOrg) are lost at the same rate as HOMs (where *k*_loss_ includes dilution, wall loss and condensation on particles) so that *ξ*_HOMs_ = [HOMs]/[OxOrg].

Particle size distribution was measured by a commercial nano-SMPS (TSI 3938) with a water-condensation particle counter (CPC; TSI 3788) for 4.6–60.0 nm, by a custom-built SMPS, with a long differential mobility analyser (DMA) and a CPC; TSI 3010) for 20–400 nm, and by a DMA train^34^, which consists of six DMAs and CPCs in parallel for 1.8–8.0 nm.

Vapour wall losses in the chamber were characterized using sulfuric acid decay experiments^35^. The OH concentration was derived from the sulfuric acid concentration using *k*_SA_ = 8.6 × 10^–13^ molecule^–1^ cm^3^ s^–1^ taken from Master Chemical Mechanism^36,37^ and cross-checked with the amount of trimethylbenzene reacted, which agreed within 20% (Extended Data Fig. 1)^36,37^. Time series of aromatic first-generation oxidation products were calculated by

1 $$\frac{{\mathrm{d}}\left[{\mathrm{G}}1\right]}{{{\mathrm{d}}t}}={k}_{{{\mathrm{VOC}}}}\left[{{\mathrm{OH}}}\right]\left[{{\mathrm{ArHC}}}\right]-{k}_{{{\mathrm{loss}}}}[{\mathrm{G}}1]$$

where [G1], [OH] and [ArHC] are the concentrations of the first-generation oxidation products, OH and aromatic precursors, respectively.

Losses of oxidation products (*k*_loss_) include dilution (*k*_dil_ = 1.6 × 10^–4^ s^–1^), wall loss (*k*_wall_ = 0.0014 s^–1^), the condensation sink (CS) to particles in the chamber, and further reaction with OH (*k*_G1_ = 4.7 × 10^–11^ molecule^–1^ cm^3^ s^–1^. The last is the OH reaction-rate constant of cresol, an important first-generation oxidation product of toluene, which was selected as surrogate for OH reaction rates of first-generation products.

2 $${k}_{{{\mathrm{loss}}}}=\left({k}_{{{\mathrm{dil}}}}+{k}_{{{\mathrm{wall}}}}+{{\mathrm{CS}}}+{k}_{{\mathrm{G}}1}[{{\mathrm{OH}}}]\right)$$

Besides the loss of first-generation products by OH reaction, their loss rates range from *k*_loss_ = (*k*_dil_ *+* *k*_wall_ + CS) when dilution, wall loss and condensation to particles in the chamber occur, to *k*_loss_ *=* *k*_dil_ when dilution is the only loss process, for example, for highly volatile, moderately reactive compounds.

### Assignment of first- and second-generation products

We illustrate typical trends of first- and second-generation oxidation products from OH-initiated oxidation of aromatics following a simplified mechanistic scheme considering RO_2_ initiated by OH oxidation and terminated via RO_2_ + NO, RO_2_ + HO_2_ or RO_2_ + RO_2_ reaction channels using the rate constants given in Extended Data Table 1.

Second-generation oxidation products are modelled with a modelled first-generation compound that reacts with OH with a reaction-rate constant *k*_G1_ = 4.7 × 10^–11^ molecule^–1^ cm^3^ s^–1^ based on cresol’s OH reactivity taken from the Master Chemical Mechanism^36,37^. See Supplementary Information for detailed steps.

On the basis of their time series, oxygenated organics are grouped into five groups (Extended Data Fig. 4) based on the correlation to modelled time series: (1) first-generation products with dilution lifetime (G1_d_), (2) first-generation products with wall-loss lifetime (G1_w_), (3) second-generation products with dilution lifetime produced from first-generation products with dilution lifetime (G2_dd_), (4) second-generation products with wall-loss lifetime produced from first-generation products with dilution lifetime (G2_dw_) and (5) second-generation products with wall-loss lifetime produced from first-generation products with wall-loss lifetime (G2_ww_).

The yields of the *i*^th^ first-generation gas-phase molecules are determined as *γ*_G1,*i*_ = [OxOrg_G1,*i*_]/G1 and the yields of the *i*^th^ second-generation gas-phase molecules are determined as *γ*_*G2,i*_ = [OxOrg_G2,*i*_]/G2 where G1 and G2 are calculated on the basis of corresponding loss rates.

### Volatility basis sets and particle growth

The volatilities of organic compounds expressed by their saturation vapour pressure (C*) are parameterized on the basis of their molecular formula as described in Supplementary Information (Volatility basis sets), Supplementary Fig. 7 and ref. ^21^. Particle growth is modelled using the gas-phase concentration and volatility of the measured oxidation products according to the VBS framework^38^, using an average molecular weight of 200 Da for organics, a Kelvin diameter of 4.8 nm and a sticking coefficient *a* = 1 (ref. ^39^). Details of the model can be found in the Supplementary information (Particle growth).

### Error analysis

Errors in our analysis of SOA yields include errors on yield calculations for first- and second-generation products and their volatility, log C*.

### Yields for first- and second-generation products

Yields for first- and second-generation products are calculated as *γ*_G1,*i*_ = [OxOrg_G1,*i*_]/G1 and *γ*_G2,*i*_ = [OxOrg_G2,*i*_]/G2, respectively, where G1 and G2 are calculated on the basis of corresponding loss rates, including dilution, wall loss and condensation onto particles. In the following, we discuss the errors related to the yield determination.

The quantification of [OxOrg] is subject to two types of error.

- Precision errors and systematic biases related to high-resolution mass spectra fitting. Precision errors have negligible effects on the analysis as we rely on averaged concentrations across an entire experiment of a few hours. Systematic biases result mainly from inaccuracies in *m/z* calibrations and relate mainly to minor shoulder or sandwich peaks that have little influence on the yields. We note that the use of single precursors during experiments results in relatively clean mass spectra. We also note that error in the assignments of peaks to an individual formula has no effect on our analysis as it is based on time-series correlations to determine first- versus second-generation products.
- Systematic errors in ionization efficiencies of the NO_3_-CIMS and PTR3 towards OxOrgs. We used the comparison of the concentrations of compounds detected (O_5_–O_6_) in both instruments to infer the accuracy of our measurements. The agreement between the two instruments is within 50% for single components. However, we note that at least ten compounds are lumped together within each volatility bin, which results in an average relative error in the concentration of each OxOrg volatility bin of ~16%. Errors related to compound assignments to different classes depend on the correlation of their time series with modelled time series. The correlation analysis indicates that over 90% of the compounds were unambiguously assigned to corresponding classes, showing statistically significantly higher correlation with the corresponding modelled time series. In case of ambiguity, compounds were assigned to first-generation products, which means that the contribution of second-generation products to SOA presented here are lower estimates.

Errors related to the determination of G1 and G2 include the following:

- Errors associated with the quantification of OH concentrations based on sulfuric acid concentrations. We compared these OH concentrations with those we determined using trimethylbenzene as a tracer to infer the accuracy of our estimates to be within 4% (Extended Data Fig. 1). As G1 and G2 have a linear and squared dependence on OH, respectively, this translates into relative errors of 4% and 16%, respectively. We note, however, that these errors affect in the same way G1 and G2, and hence *γ*_G1,*i*_ and *γ*_G2,*I*_, and thus have little to no effect on the relative importance of first- versus second-generation products.
- Errors associated with the determination of the compounds’ loss rates. We separate compounds into two categories on the basis of their loss either to the walls or by dilution. We show that the NO_3_-CIMS detects mostly LVOCs and ELVOCs that are irreversibly lost to the wall (Extended Data Fig. 2). The wall-loss rate used is centred around the average HOM loss rates with a geometric standard deviation of 1.4. This means that yields of single LVOCs and ELVOCs may vary within a factor of 1.4 while errors on lumped volatility bins are ~31%, but with no systematic biases. The error on the concentration of a single volatility bin is obtained by assuming an average of ten compounds per volatility bin and a normal distribution of the errors. Meanwhile, the PTR3 detects first-generation SVOCs and IVOCs that are lost by dilution or to the wall with varying sticking coefficients (Extended Data Fig. 2). Yields are calculated with loss rates of individual compounds to minimize errors associated with compound loss-rate variations. Loss rates of individual compounds are determined as median values of loss rates in decay stages with particle condensation less than 0.002 s^–1^ when UV radiation was turned off. For SVOCs and IVOCs, sticking coefficients may vary due to wall conditioning, but this effect will cancel out because we also take median values of yields from different stages. In the case of G2 compounds, we separate them into two categories on the basis of the loss of the G1 compounds they are produced from. We could clearly separate G2 compounds produced from G1 lost to the walls (0.0014 s^–1^) and lost by dilution (0.00016 s^–1^) on the basis of the appearance time. Uncertainties based on the variations in the measured loss rates of G1 translate into 20% relative error in the yields of G2.
- Errors associated with the assumed reaction-rate coefficient between OH and first-generation products. Here we assume the OH reaction-rate coefficient of cresol of 4.7 × 10^–11^ molecule^–1^ cm^3^ s^–1^ as a proxy for first-generation products. This rate coefficient is similar to the average rate coefficient of first-generation products reported in the Master Chemical Mechanism of 5.7 × 10^–11^ molecule^–1^ cm^3^ s^–1^ (first quartile = 1.7 × 10^–11^ molecule^–1^ cm^3^ s^–1^; third quartile = 7.9 × 10^–11^ molecule^–1^ cm^3^ s^–1^). We have conducted a sensitivity analysis using the quartile rates to assess the sensitivity of the yields on the assumed rate coefficients. The results are shown in Supplementary Fig. 8 for C_7_H_10_O_8_ as an example, which is a G2 formed from a G1_W_ through HO_2_ or RO_2_ pathway. A reaction-rate coefficient of 1.7 × 10^–11^ molecule^–1^ cm^3^ s^–1^ gives a 150% higher yield, while a reaction-rate coefficient of 7.9 × 10^–11^ molecule^–1^ cm^3^ s^–1^ gives a 35% lower yield compared with a coefficient of 4.65 × 10^–11^ molecule^–1^ cm^3^ s^–1^ (Supplementary Figs. 8 and 9). We note that these errors relate to the determination of absolute yields of single G2 products. However, since the reaction-rate coefficients of the many compounds contributing to the cumulative yield are expected to vary in this range, the average error is relatively small (~15%). We also note that in chemical transport models, the choice of a specific reaction-rate coefficient has limited effect on the results, provided that the same reaction-rate coefficient is used to determine the G2 yields.
- Isomers: One formula may contain more than one isomer from different reaction pathways. G1 and G2 separation and yields are determined for NO_*X*_ and non-NO_*X*_ pathways separately to minimize the uncertainty due to a change of dominating reaction pathways.

### Uncertainties on the determination of the compounds’ volatility

Uncertainties on the determination of the compounds’ volatility directly affect SOA yields at different OA concentrations. Fragmentation may occur when detecting organic compounds with chemical ionization, especially N-containing oxidation products may lose –NO_2_ functional groups when detected with a PTR3. However, losing an –NO_2_ functional group has little to no effect on the volatility parameterization (the effect on volatility of –ONO_2_ is roughly equivalent to –OH (refs. ^40,41^)). The volatility measurements by FIGAERO-CIMS are described in ref. ^21^. From those measurements, a VBS parameterization was derived, which was used here to determine C* of all measured compounds. Compounds showing only one *T*_max_ are used to minimize the effects of compound thermal decomposition on volatility determination. The resulting parameterization fits equally well first- and second-generation compounds, with an average precision error of 1 decade in C* for single compounds (a factor of 3 per volatility bin). Such non-systematic errors have no to limited influence on the relative difference in the yields of first- versus second-generation products.

Overall, errors on the determination of single-compound yields and their volatilities are large, factors of ~3.5 (from error propagations) and ~10, respectively. However, errors on lumped volatility bins and especially on cumulative yields, which are important for our conclusions, are much smaller—within factors of 3 and 1.8 for single volatility bins. Further, none of the errors is systematic and none affects the first- and second-generation products in a substantially different manner. Average errors on the yields of individual compounds estimated on the basis of experimental variability are around 35% (12% for each volatility bin), as shown in Supplementary Tables 1–12. We have obtained closure between measured and modelled growth rates and SOA mass, the latter based on compound volatilities and yields. This is a direct and independent assessment of the accuracy of our estimation of compound yield and volatility. Overall, errors in our analysis do not affect our conclusions giving the magnitude of the effect of second-generation chemistry on the increase in ELVOC, LVOC and SOA yields, which is an increase by up to a factor of 10 in yield or a decrease by several orders of magnitude in volatility.

### Air quality modelling

The yields of the first- and second-generation condensable gases were implemented in the Comprehensive Air Quality Model with Extensions version 6.5 to investigate the effects on regional air quality modelling. Entire-year simulations in 2011 were performed over Europe (15° W–35° E, 35°–70° N) with a resolution of 0.25° × 0.125° and 14 vertical layers from ~20 m above ground level to 460 hPa. A 1.5-dimensional VBS scheme^42^ was adopted to simulate the formation and evolution processes of organic aerosols. To separate the contributions of first- and second-generation reactions to SOA, we modified the VBS module to include eight sets, including emitted primary organic aerosols (POA) from anthropogenic activities (particle anthropogenic primary; PAP) and biogenic sources (particle biogenic primary; PBIP), oxygenated OA from POA (particle anthropogenic secondary; PAS), biogenic sources (particle biogenic secondary; PBIS), the first-generation reaction of VOC (PA1VS) and IVOC (PA1IS), and the second-generation products of VOC (PA2VS) and IVOC (PA2IS). We increased the number of VBS bins from 5 (C* = [10^–1^, 1, 10, 10^2^, 10^3^] μg m^–3^) to 6 (C* = [10^–1^, 1, 10, 10^2^, 10^3^, 10^4^] μg m^−3^) to include more volatile gases. On the basis of the fitted yields under high and low NO_*x*_ conditions for the first- and second-generation products of VOCs and IVOCs, we modified the yield parameters of benzene (BNZ), toluene (TOL), xylene (XYL) and IVOC in the VBS model. The fitted yields of toluene were applied to TOL and BNZ, and yields of trimethylbenzene and naphthalene were applied to XYL and IVOC, respectively. The products of BNZ, TOL and XYL were then merged to PA1VS (first generation) and PA2VS (second generation), while the products of IVOC went to PA1IS and PA2IS. For other sets, the default yield parameters were used. On the basis of our previous study^29^, the IVOC emissions from biomass burning were calculated as 12 times POA emissions. Population-density-weighted probability density function of ASOA_2nd to AOA was obtained using Gridded Population of the World, version 4 datasets^43^. A base case using default parameters for all the sets was developed for further comparison^44^. See Supplementary Information (Air quality model performance) for model performance^44^.

## Online content

Any methods, additional references, Nature Portfolio reporting summaries, source data, extended data, supplementary information, acknowledgements, peer review information; details of author contributions and competing interests; and statements of data and code availability are available at 10.1038/s41561-025-01645-z.

## Supplementary information

Supplementary Information Supplementary information of materials and methods, Figs. 1–9 and Tables 1–13.

## Figures and Tables

**Extended Data Table 1:**
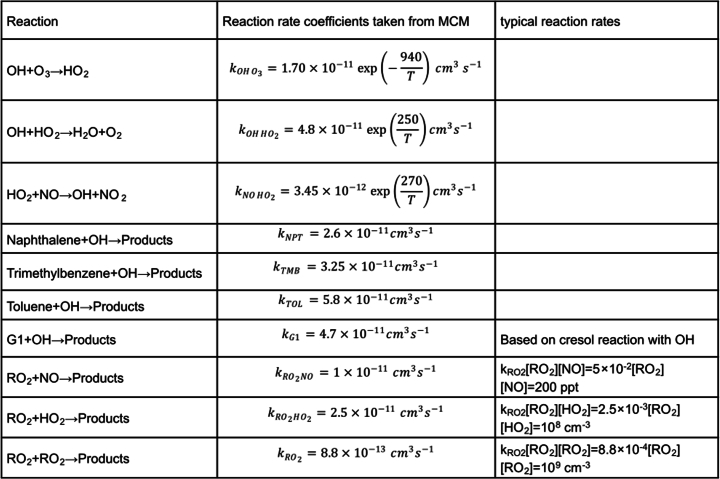
Reaction rate coefficients

**Extended Data Table 2:**
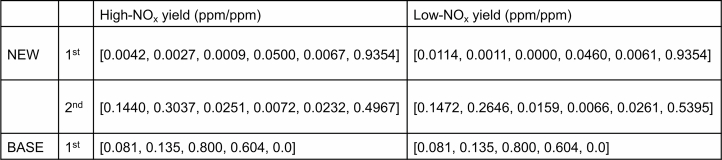
Yield parameters for anthropogenic IVOCs used in this study

**Extended Data Table 3:**
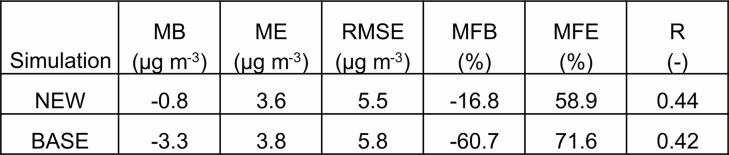
Statistical results for modeled and measured OA at 9 sites in 2011 (Nsample = 575)

MB: mean bias, ME: mean error, RMSE: root mean square error, MFB: mean fractional bias, MFE: mean fractional error.R: pearson correlation coefficient.

## Data Availability

The dataset shown in the figures and tables is available via Figshare (10.6084/m9.figshare.28099418) (ref. ^45^). Gridded Population of the World, version 4 (GPWv4) datasets^43^ used for PDF are from 10.7927/H4JW8BX5.

## Code availability

The standard CAMx model (version 6.5) is an open source model and free to download at http://www.camx.com/. The modified module for VBS with separated contributions of first- and second-generation reactions to SOA is available via Zenodo (10.5281/zenodo.14616237) (ref. ^46^).
