# Supplementary material for: Anthropogenic organic aerosol in Europe produced mainly through second-generation oxidation
Source: Nat Geosci. 2025 Mar 10;18(3):239–45. doi: 10.1038/s41561-025-01645-z (PMC11903316; doi:10.1038/s41561-025-01645-z)
Supplement: Supplementary file 1 — Supplementary information of materials and methods, Figs. 1–9 and Tables 1–13. [file 41561_2025_1645_MOESM1_ESM.pdf]

nature geoscience

Supplementary information

https://doi.org/10.1038/s41561-025-01645-z

Anthropogenic organic aerosol in Europe produced mainly through second-generation oxidation

In the format provided by the authors and unedited

Table of Contents

Supplementary information of materials and methods
Supplementary Table S1-S13
Supplementary Figure S1-S9

Methods

HOMs yields determination

To determine the apparent HOMs yield (ξ_{HOMs}), it is assumed that the oxidation products from aromatic precursors (OxOrg) are lost at the same rate as HOMs (where k_{loss} includes dilution, wall loss and condensation on particles) so that ξ_{HOMs}=[HOMs]/[OxOrg].

HOMs production can be described as

$$\frac{d[HOMs]}{dt} = P_{HOM} - k_{loss}[HOMs] \quad [1]$$

So

$$\frac{\xi_{HOMs} d[OxOrg]}{dt} = \xi_{HOMs} k_{VOC}[ArHC][OH] - k_{loss}\xi_{HOMs}[OxOrg] \quad [2]$$

Here, ξ_{HOMs} can be cancelled out and [OxOrg] is calculated by solving the differential equation. Then ξ_{HOMs} is determined as the slope between measured HOMs concentration and calculated OxOrg concentration.

Vapor wall losses characterization

Vapor wall losses in the chamber were characterized using sulfuric acid decay experiments. Loss rates of particles to the chamber wall were scaled from the sulfuric acid wall loss rate and confirmed by decay experiments of ammonium sulfate particles[1]. The lifetime of oxygenated organics was extrapolated from sulfuric acid assuming a molecular weight of 200 Da. The lifetimes of individual compounds were also determined from their decay rates during the light-off stages at the end of an experiment and used as inputs for checking their appearance time.

OH determination

The OH concentration was derived from the sulfuric acid concentration and cross-checked with the amount of trimethylbenzene (TMB) reacted. To determine OH from the sulfuric acid concentration, first sulfuric acid change rates were fitted with a 15 min time window. A sulfuric acid production rate, P_{SA}, was then obtained by considering the sulfuric acid losses to the chamber wall and to particles. The OH concentration was determined as:

$$[OH] = \frac{P_{SA}}{k_{SA}[SO_2]} \quad [3]$$

where k_{SA}=8.6 × 10^{-13} cm^3 s^{-1} is taken from the Master Chemical Mechanism[2,3].

The OH concentration was also determined by fitting the TMB time series using the following equation:

$$\frac{d[TMB]}{dt} = k_{inj} - k_{dil} - k_{TMB}[OH][TMB] \quad [4]$$

where k_{inj} is the injection rate of TMB and k_{dil} is the chamber dilution rate. The reaction rate constant for OH and TMB (k_{TMB}) is 3.25 × 10^{-11} cm^3 s^{-1}. OH concentrations calculated from the two methods agreed within 20% (Extended Data Fig. 1).

Assignment of first- and second-generation products

We developed an approach to discriminate first- and second-generation oxidation products from OH initiated oxidation of aromatics, considering their reaction with OH produces RO_2, which then terminates via RO_2+NO, RO_2+HO_2 or RO_2+RO_2 reaction channels. Since the oxidation products are formed via different reaction channels and have different loss rates, we

use a simplified mechanistic scheme to illustrate their typical trends. The following reactions are considered:

$$ArHC + OH \xrightarrow{k_{ArHC}} RO_{2,G1}$$

$$RO_{2,G1} + NO \xrightarrow{k_{RO_2NO}} G1NO$$

$$RO_{2,G1} + RO_2 \xrightarrow{k_{RO_2}} G1RO_2$$

$$RO_{2,G1} + HO_2 \xrightarrow{k_{RO_2HO_2}} G1HO_2$$

Second-generation oxidation products are modelled similar to the first-generation but RO_2 radicals are produced from OH reacting with a modelled first-generation compound using a reaction rate constant k_{G1}= 4.7×10^{-11} molecule^{-1} cm^3 s^{-1} based on cresol's OH reactivity taken from the master chemical mechanism, MCM[2,3].

$$G1 + OH \xrightarrow{k_{G1}} RO_{2,G2}$$

$$RO_{2,G2} + NO \xrightarrow{k_{RO_2NO}} G2NO$$

$$RO_{2,G2} + RO_2 \xrightarrow{k_{RO_2}} G2RO_2$$

$$RO_{2,G2} + HO_2 \xrightarrow{k_{RO_2HO_2}} G2HO_2$$

$RO_{2,G1}$ and $RO_{2,G2}$ are the peroxy radicals (RO_2) produced from OH oxidation of aromatic hydrocarbons and first-generation products, respectively. G1NO, G1RO_2, G1HO_2, G2NO, G2RO_2 and G2HO_2 are the products of $RO_{2,G1}$ and $RO_{2,G2}$ terminated by NO, RO_2 and HO_2, respectively. The following equations apply based on the above reaction scheme.

$$[RO_2] = [RO_{2,G1}] + [RO_{2,G2}] \quad [5]$$

$$\frac{d[HO_2]}{dt} = \gamma_{HO2} k_{ArHC}[ArHC][OH] + k_{OHO3}[OH][O_3] + k_{OHCO}[CO][OH] - k_{OHHO2}[OH][HO_2] - k_{NOHO2}[NO][HO_2] - k_{RO2HO2}[RO_2][HO_2] - k_{HO_2}[HO_2]^2 \quad [6]$$

$$\frac{d[RO_{2,G1}]}{dt} = k_{ArHC}[OH][ArHC] - k_{RO_2}[RO_2][RO_{2,G1}] - k_{RO_2HO_2}[HO_2][RO_{2,G1}] - k_{RO_2NO}[NO][RO_{2,G1}] \quad [7]$$

$$\frac{d[G1NO]}{dt} = k_{RO_2NO}[RO_{2,G1}][NO] - (k_{loss} + k_{G1}[OH])[G1NO] \quad [8]$$

$$\frac{d[G1HO_2]}{dt} = k_{RO_2HO_2}[RO_{2,G1}][HO_2] - (k_{loss} + k_{G1}[OH])[G1HO_2] \quad [9]$$

$$\frac{d[G1RO_2]}{dt} = k_{RO_2}[RO_{2,G1}][RO_2] - (k_{loss} + k_{G1}[OH])[G1RO_2] \quad [10]$$

$$[G1] = [G1RO_2] + [G1HO_2] + [G1NO] \quad [11]$$

$$\frac{d[RO_{2,G2}]}{dt} = k_{G1}[OH][G1] - k_{RO_2}[RO_2][RO_{2,G2}] - k_{RO_2HO_2}[HO_2][RO_{2,G2}] - k_{RO_2NO}[NO][RO_{2,G2}] \quad [12]$$

$$\frac{d[G2NO]}{dt} = k_{RO_2NO}[RO_{2,G2}][NO] - k_{loss}[G2NO] \quad [13]$$

$$\frac{d[G2HO_2]}{dt} = k_{RO_2HO_2}[RO_{2,G2}][HO_2] - k_{loss}[G2HO_2] \quad [14]$$

$$\frac{d[G2RO_2]}{dt} = k_{RO_2}[RO_{2,G2}][RO_2] - k_{loss}[G2RO_2] \quad [15]$$

The equations are solved numerically with the rate constants given in Table 1.

In the simplified model, 100 ppbv CO converts OH to HO_2 at k_{OHCO}=2.175×10^{-13} cm^3 s^{-1}, accounting for this impurity in synthetic air; pathways of HO_2 sources such as the formation of phenolic compounds as well as RO radicals are not considered separately. Instead, γ_{HO2}=1 is used to account for HO_2 generation from the reactions of aromatic precursors. This approach will not reproduce HO_2 accurately. However, we aim not to present the absolute concentrations

but the relative time trends of the oxidation products. For example, oxidation products formed through termination of the peroxy radicals $RO_{2,G1}$ and $RO_{2,G2}$ by another peroxy radical exhibit a non-linear dependence on reacted aromatics as RO_2 production increases with an increase in their reaction rates with OH (Fig. S1). For NO termination reactions a nearly linear dependence is expected. Under our experimental conditions the peroxy radical lifetime is determined by NO_x (see Table 1 and Fig. 1B) but not by RO_2 and HO_2. Therefore, the resulting modelled time series are not highly sensitive to the modelled absolute concentrations of RO_2 and HO_2, and are expected to present the relative trends in the products, which is sufficient to be used to identify first- and second-generation products.

Oxygenated organics are grouped into five groups (Extended data Fig. 4) based on the correlation to modelled time series of different dominant production and loss processes: (1) first-generation products with dilution lifetime ($G1_d$); (2) first-generation products with wall-loss lifetime ($G1_w$); (3) second-generation products with dilution lifetime produced from first-generation products with dilution lifetime ($G2_{dd}$); (4) second-generation products with wall-loss lifetime produced from first-generation products with dilution lifetime ($G2_{dw}$) and (5) second-generation products with wall-loss lifetime produced from first-generation products with wall-loss lifetime ($G2_{ww}$) .

First, we separate the oxidation products into a dilution loss and wall loss group, based on their decay rates after UV lights off. Decay rates are fitted with an exponential function after OH production is shut down (by turning off the UV lights).

After the initial separation, we group the compounds into the five categories based on the correlation to modelled time series. The appearance time is determined by fitting the function in Equation (16) to the time evolution of a compound.

$$[C] = \frac{a-b}{1+\left(t/t_{app}\right)^{a}} + b \quad [16]$$

Here a and b represent the plateau and background values of gas concentrations during the experimental stage. The appearance time is determined as the time when the 50% value between the plateau and background is reached. Time sequences are modelled for each group assuming 100% of total oxidation products are in the group using production and loss rates of the group.

The yields of the ith first-generation gas-phase molecules are determined as $\gamma_{G1,i}$=[$OxOrg_{G1,i}$]/G1 and the yields of the ith second-generation gas-phase molecules are determined as $\gamma_{G2,i}$=[$OxOrg_{G2,i}$]/G2 where G1 and G2 are calculated based on corresponding loss rates including dilution, wall loss and condensation onto particles. The reaction of G1 with OH is also considered as a sink.

Volatility basis sets

The volatilities of organic compounds expressed by their saturation vapor pressure (C^*) are parameterized based on their molecular formula as described in Wang et al. [4]:

$$\log_{10} C^*_{300} = \left(n_C^0 - n_c\right)b_c - n_o b_o \quad [17]$$

Where C^*_{300} is the saturation vapor pressure at 300 K in µg m^{-3}; n_c is the carbon number in the molecule and $n_c^0 = 25$ based on the reference carbon number of pure hydrocarbons with C^*= 1 µg m^{-3}, where each carbon addition decreases $\log_{10} C^*_{300}$ on average by $b_c = 0.48$.

Additional functional groups also decrease $\log_{10} C^*_{300}$ and are parameterized by the effective oxygen number (n_o) in the formula. A nitrate group ($-ONO_2$) typically reduces $\log_{10} C^*_{300}$ by 2.5 and for simplicity $–ONO_2$ is counted as –OH. So n_o is the oxygen number subtracted by twice the nitrogen number. For oxygenated organic compounds originating from aromatic hydrocarbon oxidation, b_o=1.72 is applied in the model based on FIGAERO measurements [4].

Particle growth

Particle growth is modelled based on the gas-phase concentration and volatility of the measured oxidation products. Compounds measured in the gas phase by PTR3 and NO_3-CIMS are grouped into volatility bins according to the VBS framework[5]. If a molecular formula is detected by both PTR3 and NO_3-CIMS, its concentration is taken from NO_3-CIMS when it contains more than 6 oxygen atoms in its formula, otherwise its concentration is taken from PTR3.

The driving force of gas to particle partitioning is described as:

$$F_i = C_{g,i} - C_{eq,i} \qquad [18]$$

where $C_{g,i}$ is the concentration of the ith VBS bin in the gas phase and $C_{eq,i}$ is the equilibrium concentration of the ith VBS bin where $C_{eq,i} = \chi_i K_p C^*_p$. χ_i is the activity, whereby the particle is simplified as ideal solution, so $\chi_i = \frac{C_{p,i}}{C_{p,tot}}$, the ratio of the ith bin in the particle phase ($C_{p,i}$) to the total concentration of all molecules in the particle phase ($C_{p,tot}$). K_p accounts for the curvature effect of the particle surface $K_p = 10^{\frac{KD \times M}{T \times D_p}}$. D_p is the particle diameter. Here, an average molecular weight of 200 Da is used for organics (M=200). A Kelvin diameter KD of 4.8 nm is used as in previous work[6].

The gas to particle condensation flux can be described as:

$$\Phi_i = K_i F_i \qquad [19]$$

A monodisperse population of nucleated particles of 1.8 nm is prescribed to model particle growth in size. The seed particles are assumed to be made of pure ammonium sulfate. K_i is the collision rate coefficient between particle and gas molecule:

$$K_i = a_i \left(\frac{3}{4\pi}\right)^{\frac{1}{6}} \left(\frac{6k_bT}{m_i} + \frac{6k_BT}{m_p}\right)^{\frac{1}{2}} \left(V_i^{\frac{1}{3}} + V_p^{\frac{1}{3}}\right)^2 \qquad [20]$$

Where k_B is the Boltzmann constant, T is the temperature, m_i, m_p, V_i, V_p are the mass and volume of the gas molecule and particle. A sticking coefficient $a_i = 1$ is used here.

Mass or volume growth is modelled for aerosols above 6 nm in diameter (the mass below 6 nm is negligible). Above that size the Kelvin effect is ignored in the model and the condensation rate K_i is approximated by the condensation sink of the aerosol population.

$$K_i = \sum 2\pi\beta D N_p D_p \qquad [21]$$

where $\beta = \frac{Kn+1}{(0.377Kn)+1+\left(\frac{4}{3a_i}\right)Kn^2+\left(\frac{4}{3a_i}\right)Kn}$ is the correction factor for non-continuum dynamics; K_n is the Knudsen number; D is the diffusion coefficient of the gas molecule; N_p is the number concentration of particles at diameter D_p.

Thus, the volume growth of aerosol can be described as:

$$\frac{dV}{dt} = \sum \Phi_i V_i - (k_{dil} + k_{wall})V \qquad [22]$$

where V is the total particle volume; k_{dil} and k_{wall} account for particle dilution and wall loss.

Solving the equations describing the gas particle partitioning process yields the growth of aerosols in diameter and volume.

Air Quality Model Performance

The modeled performance on predicting the concentrations of the major air pollutants (O_3, NO_2, SO_2, $PM_{2.5}$) was evaluated by comparing them to the measurements from the European Environment Agency database, AirBase v7, and OA measurements from 9 ACSM/AMS stations over Europe (Extended Data Fig. 7). Compared to the base case, the new parameters improve the model performance on OA. The mean bias (MB) between the modeled and measured OA is reduced from -3.3 μg m^{-3} to -0.8 μg m^{-3}, and the mean fractional bias (MFB) is reduced from -60.7% to -16.8% by using the new parameters.

Reference:

1. Stolzenburg, D. *et al.* Enhanced growth rate of atmospheric particles from sulfuric acid. *Atmos Chem Phys* **20**, 7359–7372 (2020).
2. Bloss, C. *et al.* Development of a detailed chemical mechanism (MCMv3.1) for the atmospheric oxidation of aromatic hydrocarbons. *Atmos Chem Phys* **5**, 641–664 (2005).
3. Jenkin, M. E., Saunders, S. M., Wagner, V. & Pilling, M. J. Protocol for the development of the Master Chemical Mechanism, MCM v3 (Part B): tropospheric degradation of aromatic volatile organic compounds. *Atmos Chem Phys* **3**, 181–193 (2003).
4. Wang, M. *et al.* Photo-oxidation of aromatic hydrocarbons produces low-volatility organic compounds. *Environ Sci Technol* **54**, 7911–7921 (2020).
5. Donahue, N. M., Kroll, J. H., Pandis, S. N. & Robinson, A. L. A two-dimensional volatility basis set-Part 2: Diagnostics of organic-aerosol evolution. *Atmos Chem Phys* **12**, 615–634 (2012).
6. Stolzenburg, D. *et al.* Rapid growth of organic aerosol nanoparticles over a wide tropospheric temperature range. *Proc Natl Acad Sci U S A* **115**, 9122–9127 (2018).

Supplementary Table S1 Compound formulas measured by the CIMS, average yields and associated standard deviations (variation based on different runs) for toluene OH oxidation under no NO_x conditions. Compounds are distinguished between first- and second- generation products.

Formula	C	H	O	N	LogC^*	Yield	std	Generation
C5H8O5	5	8	5	0	1	2.1E-03	2.1E-04	G1
C5H6O8	5	6	8	0	-4.16	7.4E-05	9.1E-06	G1
C6H6O4	6	6	4	0	2.24	1.2E-04	1.9E-05	G1
C6H8O5	6	8	5	0	0.52	2.0E-04	2.2E-05	G1
C6H8O6	6	8	6	0	-1.2	4.8E-04	3.1E-05	G1
C6H10O6	6	10	6	0	-1.2	2.0E-04	3.5E-05	G1
C6H8O7	6	8	7	0	-2.92	7.4E-04	7.0E-05	G1
C6H10O7	6	10	7	0	-2.92	1.1E-04	1.3E-05	G1
C6H8O8	6	8	8	0	-4.64	5.4E-04	7.6E-05	G1
C6H9O9N1	6	9	9	1	-2.92	1.4E-05	8.0E-07	G1
C6H9O10N1	6	9	10	1	-4.64	2.1E-05	2.7E-06	G1
C7H8O4	7	8	4	0	1.76	6.9E-04	6.6E-05	G1
C7H10O4	7	10	4	0	1.76	6.9E-04	1.0E-04	G1
C7H6O6	7	6	6	0	-1.68	4.0E-04	5.8E-05	G1
C7H8O6	7	8	6	0	-1.68	8.7E-04	9.5E-05	G1
C7H8O7	7	8	7	0	-3.4	5.1E-04	7.2E-05	G1
C7H8O8	7	8	8	0	-5.12	9.4E-04	8.4E-05	G1
C7H8O9	7	8	9	0	-6.84	7.7E-04	1.0E-04	G1
C7H10O9	7	10	9	0	-6.84	6.0E-04	4.7E-05	G1
C7H8O10	7	8	10	0	-8.56	6.5E-04	1.1E-04	G1
C7H10O10	7	10	10	0	-8.56	5.2E-04	6.6E-05	G1
C7H8O11	7	8	11	0	-10.28	2.8E-04	5.5E-05	G1
C13H18O9	13	18	9	0	-9.72	6.4E-05	5.0E-06	G1
C13H18O11	13	18	11	0	-13.16	3.3E-05	3.4E-06	G1
C14H18O6	14	18	6	0	-5.04	4.1E-06	3.2E-06	G1
C14H16O7	14	16	7	0	-6.76	4.9E-05	5.0E-06	G1
C14H18O8	14	18	8	0	-8.48	4.0E-03	4.4E-04	G1
C14H16O9	14	16	9	0	-10.2	1.1E-04	2.1E-05	G1
C14H18O9	14	18	9	0	-10.2	2.2E-04	2.3E-05	G1
C14H18O10	14	18	10	0	-11.92	4.1E-04	4.2E-05	G1
C14H18O12	14	18	12	0	-15.36	4.9E-04	5.5E-05	G1
C5H7O6N1	5	7	6	1	2.72	3.2E-04	1.1E-04	G2
C5H6O7	5	6	7	0	-2.44	2.1E-03	4.4E-04	G2
C5H7O7N1	5	7	7	1	1	4.0E-04	1.4E-04	G2
C6H8O4	6	8	4	0	2.24	2.6E-03	6.0E-04	G2
C7H6O5	7	6	5	0	0.04	2.4E-03	7.2E-04	G2
C7H8O5	7	8	5	0	0.04	2.4E-02	4.4E-03	G2
C7H10O5	7	10	5	0	0.04	1.6E-01	3.4E-02	G2

C7H10O6	7	10	6	0	-1.68	3.0E-02	5.7E-03	G2
C7H10O7	7	10	7	0	-3.4	4.3E-02	9.0E-03	G2
C7H12O7	7	12	7	0	-3.4	1.0E-02	1.4E-03	G2
C7H10O8	7	10	8	0	-5.12	3.5E-02	7.6E-03	G2
C7H12O8	7	12	8	0	-5.12	6.2E-03	9.5E-04	G2
C12H16O10	12	16	10	0	-10.96	4.0E-04	8.5E-05	G2
C14H16O6	14	16	6	0	-5.04	9.6E-04	2.0E-04	G2
C14H16O8	14	16	8	0	-8.48	1.9E-03	3.8E-04	G2
C14H16O10	14	16	10	0	-11.92	1.1E-03	2.5E-04	G2
C14H20O10	14	20	10	0	-11.92	2.5E-03	3.3E-04	G2
C14H16O11	14	16	11	0	-13.64	8.3E-04	2.3E-04	G2
C14H18O11	14	18	11	0	-13.64	3.3E-03	8.0E-04	G2
C14H20O11	14	20	11	0	-13.64	3.4E-03	4.9E-04	G2
C14H16O12	14	16	12	0	-15.36	4.9E-04	1.1E-04	G2

Supplementary Table S2 Compound formulas measured by the CIMS, average yields and associated standard deviations (variation based on different runs) for toluene OH oxidation under high NO_x (>0.2 ppbv) conditions. Compounds are distinguished between first- and second-generation products.

Formula	C	H	O	N	LogC^*	Yield	std	Generation
C5H8O5	5	8	5	0	1	6.5E-04	3.7E-05	G1
C5H6O8	5	6	8	0	-4.16	4.7E-05	4.2E-06	G1
C6H5O3N1	6	5	3	1	7.4	1.6E-03	4.9E-04	G1
C6H6O4	6	6	4	0	2.24	6.0E-05	5.4E-06	G1
C6H8O5	6	8	5	0	0.52	1.1E-04	2.5E-05	G1
C6H6O6N2	6	6	6	2	5.68	1.8E-04	7.2E-05	G1
C6H8O6	6	8	6	0	-1.2	1.8E-04	2.5E-05	G1
C6H10O6	6	10	6	0	-1.2	4.4E-05	1.4E-05	G1
C6H8O7	6	8	7	0	-2.92	3.6E-04	3.4E-05	G1
C6H9O7N1	6	9	7	1	0.52	1.0E-04	5.7E-05	G1
C6H10O7	6	10	7	0	-2.92	3.5E-05	1.2E-05	G1
C6H8O8	6	8	8	0	-4.64	2.5E-04	1.9E-05	G1
C6H9O9N1	6	9	9	1	-2.92	1.0E-04	8.7E-06	G1
C6H9O10N1	6	9	10	1	-4.64	1.0E-04	2.0E-06	G1
C7H8O4	7	8	4	0	1.76	2.7E-04	8.3E-06	G1
C7H10O4	7	10	4	0	1.76	1.3E-04	6.7E-06	G1
C7H9O5N1	7	9	5	1	3.48	7.5E-04	1.3E-04	G1
C7H6O6	7	6	6	0	-1.68	7.9E-05	1.2E-05	G1
C7H8O6	7	8	6	0	-1.68	5.1E-04	8.7E-05	G1
C7H9O6N1	7	9	6	1	1.76	1.8E-03	1.9E-04	G1
C7H9O7	7	9	7	0	-3.4	1.2E-04	1.8E-05	G1
C7H9O7N1	7	9	7	1	0.04	1.3E-03	2.3E-05	G1
C7H8O8	7	8	8	0	-5.12	9.0E-04	6.0E-05	G1
C7H9O8N1	7	9	8	1	-1.68	2.7E-03	1.3E-04	G1
C7H8O9	7	8	9	0	-6.84	2.7E-04	4.1E-05	G1
C7H9O9N1	7	9	9	1	-3.4	1.1E-03	8.7E-05	G1
C7H10O9	7	10	9	0	-6.84	1.5E-04	5.2E-05	G1
C7H8O10	7	8	10	0	-8.56	2.3E-04	3.4E-05	G1
C7H9O10N1	7	9	10	1	-5.12	1.5E-03	1.5E-04	G1
C7H10O10	7	10	10	0	-8.56	6.7E-05	2.4E-05	G1
C7H9O11N1	7	9	11	1	-6.84	4.5E-04	2.9E-05	G1
C7H10O11	7	10	11	0	-10.28	2.9E-05	6.9E-06	G1
C7H9O12N1	7	9	12	1	-8.56	1.7E-04	3.0E-05	G1
C14H18O8	14	18	8	0	-8.48	9.8E-05	3.8E-05	G1
C14H16O9	14	16	9	0	-10.2	2.7E-05	1.0E-05	G1
C14H18O9	14	18	9	0	-10.2	3.1E-05	1.1E-05	G1

C14H18O10	14	18	10	0	-11.92	2.3E-05	8.6E-06	G1
C14H18O12	14	18	12	0	-15.36	4.1E-05	1.9E-05	G1
C5H7O6N1	5	7	6	1	2.72	1.0E-03	1.8E-04	G2
C5H6O7	5	6	7	0	-2.44	1.1E-03	1.2E-04	G2
C5H7O7N1	5	7	7	1	1	8.3E-04	7.5E-05	G2
C6H8O4	6	8	4	0	2.24	4.8E-04	8.1E-05	G2
C6H9O8N1	6	9	8	1	-1.2	9.7E-04	1.2E-04	G2
C7H7O3N1	7	7	3	1	6.92	1.0E-01	6.4E-02	G2
C7H7O4N1	7	7	4	1	5.2	6.0E-03	3.3E-04	G2
C7H6O5	7	6	5	0	0.04	5.5E-04	4.5E-05	G2
C7H7O5N1	7	7	5	1	3.48	1.6E-03	2.8E-04	G2
C7H8O5	7	8	5	0	0.04	6.3E-03	6.8E-04	G2
C7H10O5	7	10	5	0	0.04	3.8E-03	8.2E-04	G2
C7H7O6N1	7	7	6	1	1.76	1.5E-03	1.3E-04	G2
C7H8O6N2	7	8	6	2	5.2	4.8E-03	3.8E-03	G2
C7H10O6	7	10	6	0	-1.68	2.7E-03	5.3E-04	G2
C7H8O7N2	7	8	7	2	3.48	1.3E-03	1.7E-04	G2
C7H10O7	7	10	7	0	-3.4	1.7E-03	4.7E-04	G2
C7H12O7	7	12	7	0	-3.4	3.1E-04	7.1E-05	G2
C7H8O8N2	7	8	8	2	1.76	3.7E-04	1.0E-04	G2
C7H10O8	7	10	8	0	-5.12	2.1E-03	6.7E-04	G2
C7H10O8N2	7	10	8	2	1.76	4.1E-04	1.0E-04	G2
C7H12O8	7	12	8	0	-5.12	2.1E-04	5.5E-05	G2
C7H7O9N1	7	7	9	1	-3.4	7.2E-04	8.8E-05	G2
C7H8O9N2	7	8	9	2	0.04	7.1E-04	8.8E-05	G2
C7H8O10N2	7	8	10	2	-1.68	2.7E-04	4.0E-05	G2
C7H10O10N2	7	10	10	2	-1.68	2.8E-03	2.6E-04	G2
C7H9O11	7	9	11	0	-10.28	2.3E-04	4.5E-05	G2
C7H10O11N2	7	10	11	2	-3.4	7.7E-04	1.6E-04	G2
C7H8O12N2	7	8	12	2	-5.12	1.0E-04	1.1E-05	G2
C7H10O12N2	7	10	12	2	-5.12	3.5E-04	5.8E-05	G2
C12H16O10	12	16	10	0	-10.96	4.6E-05	1.8E-05	G2
C14H14O6N2	14	14	6	2	1.84	3.6E-03	2.2E-03	G2
C14H16O6	14	16	6	0	-5.04	1.3E-04	3.1E-05	G2
C14H16O10	14	16	10	0	-11.92	1.8E-04	5.6E-05	G2
C14H20O10	14	20	10	0	-11.92	6.0E-05	1.2E-05	G2
C14H16O11	14	16	11	0	-13.64	1.0E-04	3.7E-05	G2
C14H18O11	14	18	11	0	-13.64	1.5E-04	5.2E-05	G2
C14H20O11	14	20	11	0	-13.64	8.5E-05	2.2E-05	G2
C14H16O12	14	16	12	0	-15.36	6.3E-05	1.9E-05	G2

Supplementary Table S3 Compound formulas measured by the CIMS, average yields and associated standard deviations (variation based on different runs) for trimethylbenzene OH oxidation under no NO_x conditions. Compounds are distinguished between first- and second-generation products.

**Formula**	**C**	**H**	**O**	**N**	**LogC^***	**Yield**	**std**	**Generation**
C6H8O5	6	8	5	0	0.52	1.2E-04	2.7E-05	G1
C6H8O6	6	8	6	0	-1.2	2.3E-04	4.9E-05	G1
C7H10O7	7	10	7	0	-3.4	1.5E-03	3.9E-04	G1
C7H10O8	7	10	8	0	-5.12	5.9E-04	1.3E-04	G1
C9H12O4	9	12	4	0	0.8	2.8E-04	6.1E-05	G1
C9H12O5	9	12	5	0	-0.92	2.1E-03	3.8E-04	G1
C9H14O5	9	14	5	0	-0.92	4.9E-03	1.1E-03	G1
C9H12O6	9	12	6	0	-2.64	4.5E-03	1.0E-03	G1
C9H14O6	9	14	6	0	-2.64	2.1E-03	5.6E-04	G1
C9H12O7	9	12	7	0	-4.36	1.8E-03	3.7E-04	G1
C9H12O8	9	12	8	0	-6.08	6.7E-04	1.5E-04	G1
C9H12O9	9	12	9	0	-7.8	3.6E-04	8.5E-05	G1
C9H12O10	9	12	10	0	-9.52	2.1E-04	5.2E-05	G1
C9H12O11	9	12	11	0	-11.24	2.4E-05	5.6E-06	G1
C6H10O5	6	10	5	0	0.52	2.0E-02	6.3E-03	G2
C6H10O6	6	10	6	0	-1.2	3.3E-03	9.8E-04	G2
C6H10O7	6	10	7	0	-2.92	3.4E-03	1.1E-03	G2
C7H10O6	7	10	6	0	-1.68	2.1E-03	4.9E-04	G2
C7H10O9	7	10	9	0	-6.84	1.6E-03	6.0E-04	G2
C7H10O10	7	10	10	0	-8.56	2.2E-04	1.2E-04	G2
C8H12O6	8	12	6	0	-2.16	3.1E-03	7.4E-04	G2
C8H12O7	8	12	7	0	-3.88	6.8E-03	2.0E-03	G2
C9H10O5	9	10	5	0	-0.92	1.6E-03	7.4E-04	G2
C9H10O7	9	10	7	0	-4.36	9.5E-04	2.7E-04	G2
C9H14O7	9	14	7	0	-4.36	4.1E-02	1.3E-02	G2
C9H16O7	9	16	7	0	-4.36	1.3E-02	3.5E-03	G2
C9H14O8	9	14	8	0	-6.08	1.6E-02	4.3E-03	G2
C9H15O8N1	9	15	8	1	-2.64	1.4E-03	6.5E-04	G2
C9H16O8	9	16	8	0	-6.08	1.7E-02	5.6E-03	G2
C9H14O9	9	14	9	0	-7.8	4.2E-03	1.3E-03	G2
C9H16O9	9	16	9	0	-7.8	1.1E-03	3.0E-04	G2
C9H14O10	9	14	10	0	-9.52	1.5E-03	4.8E-04	G2
C9H16O10	9	16	10	0	-9.52	6.9E-04	1.6E-04	G2
C9H14O11	9	14	11	0	-11.24	3.4E-04	1.3E-04	G2
C9H16O11	9	16	11	0	-11.24	1.6E-04	5.4E-05	G2
C15H22O8	15	22	8	0	-8.96	1.9E-03	5.4E-04	G2
C15H22O9	15	22	9	0	-10.68	8.4E-04	2.0E-04	G2
C18H20O6	18	20	6	0	-6.96	3.8E-04	1.2E-04	G2

C18H22O7	18	22	7	0	-8.68	3.0E-04	9.2E-05	G2
C18H24O8	18	24	8	0	-10.4	4.8E-04	1.3E-04	G2

Supplementary Table S4 Compound formulas measured by the CIMS, average yields and associated standard deviations (variation based on different runs) for trimethylbenzene OH oxidation under high NO_x (>0.2 ppbv) conditions. Compounds are distinguished between first- and second- generation products.

Formula	C	H	O	N	LogC^*	Yield	std	Generation
C6H8O5	6	8	5	0	0.52	4.4E-05	3.7E-06	G1
C6H8O6	6	8	6	0	-1.2	6.5E-05	7.9E-06	G1
C7H10O7	7	10	7	0	-3.4	2.4E-04	7.2E-05	G1
C7H10O8	7	10	8	0	-5.12	1.0E-04	1.6E-05	G1
C9H12O4	9	12	4	0	0.8	6.5E-05	1.0E-05	G1
C9H12O5	9	12	5	0	-0.92	2.5E-04	3.7E-05	G1
C9H13O5N1	9	13	5	1	2.52	7.5E-04	2.5E-04	G1
C9H14O5	9	14	5	0	-0.92	2.1E-04	4.5E-05	G1
C9H12O6	9	12	6	0	-2.64	1.8E-04	7.9E-05	G1
C9H13O6N1	9	13	6	1	0.8	1.7E-03	2.5E-04	G1
C9H14O6	9	14	6	0	-2.64	1.2E-04	3.5E-05	G1
C9H12O7	9	12	7	0	-4.36	6.7E-04	5.9E-05	G1
C9H13O7N1	9	13	7	1	-0.92	5.8E-04	6.7E-05	G1
C9H12O8	9	12	8	0	-6.08	1.9E-04	2.5E-05	G1
C9H13O8N1	9	13	8	1	-2.64	1.7E-03	1.8E-04	G1
C9H14O8N2	9	14	8	2	0.8	1.3E-04	2.3E-05	G1
C9H12O9	9	12	9	0	-7.8	5.6E-05	7.4E-06	G1
C9H13O9N1	9	13	9	1	-4.36	1.2E-03	2.3E-04	G1
C9H14O9N2	9	14	9	2	-0.92	3.0E-04	4.4E-05	G1
C9H12O10	9	12	10	0	-9.52	1.8E-04	1.2E-05	G1
C9H13O10N1	9	13	10	1	-6.08	2.7E-04	2.7E-05	G1
C9H12O11	9	12	11	0	-11.24	1.2E-05	2.1E-06	G1
C6H10O5	6	10	5	0	0.52	9.3E-04	5.4E-04	G2
C6H10O6	6	10	6	0	-1.2	4.3E-04	1.7E-04	G2
C6H10O7	6	10	7	0	-2.92	7.7E-04	3.1E-04	G2
C7H10O6	7	10	6	0	-1.68	1.7E-03	9.4E-04	G2
C7H10O9	7	10	9	0	-6.84	6.5E-04	2.4E-04	G2
C7H10O10	7	10	10	0	-8.56	7.7E-04	2.2E-04	G2
C8H12O6	8	12	6	0	-2.16	1.2E-03	6.2E-04	G2
C8H12O7	8	12	7	0	-3.88	2.2E-03	9.1E-04	G2
C9H11O5N1	9	11	5	1	2.52	7.1E-04	2.8E-04	G2
C9H9O6N1	9	9	6	1	0.8	8.4E-05	4.6E-05	G2
C9H11O6N1	9	11	6	1	0.8	8.9E-04	3.7E-04	G2
C9H10O7	9	10	7	0	-4.36	1.1E-03	7.1E-04	G2
C9H11O7N1	9	11	7	1	-0.92	7.1E-04	1.9E-04	G2
C9H12O7N2	9	12	7	2	2.52	1.1E-03	3.1E-04	G2
C9H14O7	9	14	7	0	-4.36	5.7E-04	3.8E-04	G2
C9H16O7	9	16	7	0	-4.36	2.9E-04	1.1E-04	G2

C9H12O8N2	9	12	8	2	0.8	8.5E-04	1.9E-04	G2
C9H14O8	9	14	8	0	-6.08	9.9E-04	2.4E-04	G2
C9H15O8N1	9	15	8	1	-2.64	8.0E-04	3.2E-04	G2
C9H11O9N1	9	11	9	1	-4.36	5.9E-04	1.8E-04	G2
C9H12O9N2	9	12	9	2	-0.92	2.0E-03	7.9E-04	G2
C9H14O9	9	14	9	0	-7.8	3.2E-04	9.3E-05	G2
C9H15O9N1	9	15	9	1	-4.36	7.2E-04	2.3E-04	G2
C9H16O9	9	16	9	0	-7.8	2.7E-04	1.3E-04	G2
C9H12O10N2	9	12	10	2	-2.64	8.4E-04	2.5E-04	G2
C9H14O10	9	14	10	0	-9.52	2.1E-04	7.2E-05	G2
C9H14O10N2	9	14	10	2	-2.64	1.0E-02	2.6E-03	G2
C9H16O10	9	16	10	0	-9.52	1.4E-04	7.3E-05	G2
C9H12O11N2	9	12	11	2	-4.36	4.3E-04	9.6E-05	G2
C9H14O11	9	14	11	0	-11.24	1.8E-04	5.0E-05	G2
C9H14O11N2	9	14	11	2	-4.36	3.4E-03	1.2E-03	G2
C9H16O11	9	16	11	0	-11.24	7.7E-05	4.2E-05	G2
C15H22O8	15	22	8	0	-8.96	1.7E-04	5.6E-05	G2
C18H20O6	18	20	6	0	-6.96	1.3E-04	5.6E-05	G2
C18H22O7	18	22	7	0	-8.68	1.3E-04	6.9E-05	G2

Supplementary Table S5 Compound formulas measured by the CIMS, average yields and associated standard deviations (variation based on different runs) for naphthalene OH oxidation under no NO_x conditions. Compounds are distinguished between first- and second-generation products.

Formula	C	H	O	N	LogC^*	Yield	std	Generation
C9H7O8N1	9	7	8	1	-2.64	9.7E-05	1.3E-05	G1
C10H4O3	10	4	3	0	2.04	1.1E-04	1.9E-05	G1
C10H10O5	10	10	5	0	-1.4	9.1E-03	8.0E-04	G1
C10H10O6	10	10	6	0	-3.12	3.8E-03	8.1E-04	G1
C10H10O7	10	10	7	0	-4.84	2.8E-03	6.0E-04	G1
C10H12O7	10	12	7	0	-4.84	4.5E-04	8.6E-05	G1
C10H10O8	10	10	8	0	-6.56	1.9E-03	3.8E-04	G1
C10H10O9	10	10	9	0	-8.28	1.2E-03	2.8E-04	G1
C10H10O10	10	10	10	0	-10	1.1E-03	2.0E-04	G1
C7H3O5N1	7	3	5	1	3.48	1.1E-03	2.2E-04	G2
C8H4O4	8	4	4	0	1.28	1.6E-03	7.9E-04	G2
C8H6O4	8	6	4	0	1.28	5.3E-04	2.9E-04	G2
C8H7O4N1	8	7	4	1	4.72	6.1E-04	1.4E-04	G2
C8H6O5	8	6	5	0	-0.44	5.8E-04	7.6E-05	G2
C8H5O6N1	8	5	6	1	1.28	7.9E-04	3.4E-04	G2
C8H12O6	8	12	6	0	-2.16	2.6E-03	3.7E-04	G2
C8H6O7	8	6	7	0	-3.88	4.0E-04	4.0E-05	G2
C8H7O7N1	8	7	7	1	-0.44	3.5E-04	9.9E-05	G2
C8H12O7	8	12	7	0	-3.88	1.9E-03	3.1E-04	G2
C8H14O7	8	14	7	0	-3.88	1.2E-02	1.5E-03	G2
C8H6O8	8	6	8	0	-5.6	2.9E-04	8.5E-05	G2
C8H12O8	8	12	8	0	-5.6	4.2E-03	7.8E-04	G2
C9H5O5N1	9	5	5	1	2.52	4.2E-03	9.5E-04	G2
C9H8O5	9	8	5	0	-0.92	3.9E-03	4.5E-04	G2
C9H10O6	9	10	6	0	-2.64	1.3E-03	2.2E-04	G2
C9H12O7	9	12	7	0	-4.36	3.9E-04	1.1E-04	G2
C9H9O8N1	9	9	8	1	-2.64	4.9E-04	1.3E-04	G2
C9H12O8	9	12	8	0	-6.08	3.7E-04	1.1E-04	G2
C10H4O4	10	4	4	0	0.32	3.4E-03	4.1E-04	G2
C10H6O4	10	6	4	0	0.32	1.4E-03	2.7E-04	G2
C10H6O4	10	6	4	0	0.32	9.5E-03	1.6E-03	G2
C10H8O4	10	8	4	0	0.32	1.2E-02	1.8E-03	G2
C10H4O5	10	4	5	0	-1.4	5.2E-03	1.1E-03	G2
C10H5O5N1	10	5	5	1	2.04	8.2E-03	2.5E-03	G2
C10H6O5	10	6	5	0	-1.4	3.6E-03	1.3E-03	G2
C10H6O5	10	6	5	0	-1.4	3.8E-03	6.3E-04	G2
C10H7O5N1	10	7	5	1	2.04	1.6E-02	2.0E-03	G2
C10H8O5	10	8	5	0	-1.4	1.5E-02	3.0E-03	G2

C10H5O6N1	10	5	6	1	0.32	2.5E-03	3.9E-04	G2
C10H6O6	10	6	6	0	-3.12	9.6E-04	1.8E-04	G2
C10H6O6	10	6	6	0	-3.12	3.8E-03	7.0E-04	G2
C10H7O6N1	10	7	6	1	0.32	9.7E-03	1.3E-03	G2
C10H8O6	10	8	6	0	-3.12	2.0E-02	2.8E-03	G2
C10H12O6	10	12	6	0	-3.12	8.3E-03	2.1E-03	G2
C10H6O7	10	6	7	0	-4.84	6.6E-04	1.7E-04	G2
C10H6O7	10	6	7	0	-4.84	2.4E-03	2.4E-04	G2
C10H7O7	10	7	7	0	-4.84	1.5E-03	6.0E-04	G2
C10H8O7	10	8	7	0	-4.84	1.6E-02	2.3E-03	G2
C10H7O8	10	7	8	0	-6.56	4.7E-03	8.4E-04	G2
C10H8O8	10	8	8	0	-6.56	5.2E-03	6.9E-04	G2
C10H12O8	10	12	8	0	-6.56	4.5E-03	1.3E-03	G2
C10H8O9	10	8	9	0	-8.28	4.9E-03	9.8E-04	G2
C10H8O10	10	8	10	0	-10	2.6E-04	1.4E-04	G2
C10H12O10	10	12	10	0	-10	1.1E-03	2.7E-04	G2
C10H8O11	10	8	11	0	-11.72	2.4E-04	3.3E-05	G2
C10H10O11	10	10	11	0	-11.72	6.3E-04	3.4E-04	G2
C10H12O11	10	12	11	0	-11.72	7.7E-04	1.4E-04	G2

Supplementary Table S6 Compound formulas measured by the CIMS, average yields and associated standard deviations (variation based on different runs) for naphthalene OH oxidation under high NO_x (>0.2 ppbv) conditions. Compounds are distinguished between first- and second-generation products.

**Formula**	**C**	**H**	**O**	**N**	**Log*C***	**Yield**	**std**	**Generation**
C8H12O10	8	12	10	0	-9.04	3.3E-04	1.1E-04	G1
C9H7O8N1	9	7	8	1	-2.64	1.5E-04	1.8E-05	G1
C10H4O3	10	4	3	0	2.04	8.8E-05	1.4E-05	G1
C10H7O3N1	10	7	3	1	5.48	5.6E-03	1.4E-03	G1
C10H10O5	10	10	5	0	-1.4	3.7E-04	3.7E-04	G1
C10H9O6N1	10	9	6	1	0.32	3.2E-03	2.6E-04	G1
C10H10O6	10	10	6	0	-3.12	2.0E-04	1.9E-04	G1
C10H10O7	10	10	7	0	-4.84	2.0E-04	1.2E-04	G1
C10H12O7	10	12	7	0	-4.84	1.7E-05	1.0E-05	G1
C10H10O8	10	10	8	0	-6.56	9.1E-05	6.9E-05	G1
C10H9O9N1	10	9	9	1	-4.84	8.5E-04	1.0E-04	G1
C10H10O9	10	10	9	0	-8.28	6.8E-05	5.7E-05	G1
C10H10O10	10	10	10	0	-10	4.2E-05	3.3E-05	G1
C10H9O11N1	10	9	11	1	-8.28	5.3E-04	9.3E-05	G1
C7H3O5N1	7	3	5	1	3.48	7.1E-03	1.2E-03	G2
C7H5O5N1	7	5	5	1	3.48	4.5E-03	1.0E-03	G2
C8H7O3N1	8	7	3	1	6.44	2.4E-03	7.3E-04	G2
C8H4O4	8	4	4	0	1.28	1.3E-03	9.6E-04	G2
C8H6O4	8	6	4	0	1.28	1.1E-03	5.7E-04	G2
C8H7O4N1	8	7	4	1	4.72	8.4E-04	2.4E-04	G2
C8H5O5N1	8	5	5	1	3	2.2E-04	8.0E-05	G2
C8H6O5	8	6	5	0	-0.44	4.6E-04	1.2E-04	G2
C8H7O5N1	8	7	5	1	3	4.5E-04	1.3E-04	G2
C8H5O6N1	8	5	6	1	1.28	4.2E-04	8.2E-05	G2
C8H5O6N1	8	5	6	1	1.28	5.0E-04	1.2E-03	G2
C8H8O6	8	8	6	0	-2.16	2.2E-04	1.2E-04	G2
C8H8O6N2	8	8	6	2	4.72	5.8E-03	1.6E-03	G2
C8H6O7	8	6	7	0	-3.88	2.7E-04	1.9E-04	G2
C8H7O7N1	8	7	7	1	-0.44	5.3E-04	1.2E-04	G2
C8H6O8	8	6	8	0	-5.6	2.7E-04	1.1E-04	G2
C8H7O8N1	8	7	8	1	-2.16	3.4E-04	1.4E-04	G2
C8H12O8	8	12	8	0	-5.6	4.4E-04	2.4E-04	G2
C8H12O9	8	12	9	0	-7.32	2.4E-03	9.1E-04	G2
C9H5O5N1	9	5	5	1	2.52	2.6E-03	1.3E-03	G2
C9H7O5N1	9	7	5	1	2.52	5.8E-03	1.4E-03	G2
C9H8O5	9	8	5	0	-0.92	1.3E-03	5.6E-04	G2
C9H7O6N1	9	7	6	1	0.8	9.1E-04	9.2E-04	G2
C9H10O6	9	10	6	0	-2.64	1.8E-04	1.2E-04	G2

C9H9O8N1	9	9	8	1	-2.64	4.5E-04	1.2E-04	G2
C9H9O9N1	9	9	9	1	-4.36	5.4E-04	2.4E-04	G2
C10H4O4	10	4	4	0	0.32	4.7E-03	1.8E-03	G2
C10H5O4N1	10	5	4	1	3.76	3.7E-03	1.1E-03	G2
C10H6O4	10	6	4	0	0.32	7.6E-04	4.1E-04	G2
C10H6O4	10	6	4	0	0.32	1.0E-02	3.7E-03	G2
C10H7O4N1	10	7	4	1	3.76	8.6E-03	2.5E-03	G2
C10H8O4	10	8	4	0	0.32	1.4E-02	3.1E-03	G2
C10H4O5	10	4	5	0	-1.4	3.4E-03	9.3E-04	G2
C10H5O5N1	10	5	5	1	2.04	7.1E-03	4.3E-03	G2
C10H6O5	10	6	5	0	-1.4	1.1E-03	2.8E-04	G2
C10H6O5	10	6	5	0	-1.4	2.5E-03	1.0E-03	G2
C10H7O5N1	10	7	5	1	2.04	5.4E-03	2.0E-03	G2
C10H7O5N1	10	7	5	1	2.04	4.9E-03	1.2E-03	G2
C10H8O5	10	8	5	0	-1.4	1.0E-02	3.7E-03	G2
C10H9O5N1	10	9	5	1	2.04	1.0E-02	2.2E-03	G2
C10H5O6N1	10	5	6	1	0.32	1.5E-03	5.0E-04	G2
C10H6O6	10	6	6	0	-3.12	6.6E-04	1.9E-04	G2
C10H6O6	10	6	6	0	-3.12	1.3E-03	3.4E-04	G2
C10H7O6N1	10	7	6	1	0.32	1.2E-02	3.6E-03	G2
C10H8O6	10	8	6	0	-3.12	2.1E-02	7.6E-03	G2
C10H11O6N1	10	11	6	1	0.32	1.5E-03	5.9E-04	G2
C10H6O7	10	6	7	0	-4.84	3.3E-04	9.3E-05	G2
C10H6O7	10	6	7	0	-4.84	1.4E-03	6.8E-04	G2
C10H7O7	10	7	7	0	-4.84	2.1E-04	1.3E-04	G2
C10H8O7	10	8	7	0	-4.84	2.0E-02	8.2E-03	G2
C10H9O7N1	10	9	7	1	-1.4	1.2E-02	2.9E-03	G2
C10H10O7N2	10	10	7	2	2.04	6.9E-03	3.0E-03	G2
C10H11O7N1	10	11	7	1	-1.4	2.0E-03	1.0E-03	G2
C10H7O8N1	10	7	8	1	-3.12	3.3E-03	1.1E-03	G2
C10H7O8	10	7	8	0	-6.56	1.5E-03	5.4E-04	G2
C10H8O8	10	8	8	0	-6.56	5.3E-03	2.2E-03	G2
C10H8O8N2	10	8	8	2	0.32	6.9E-04	2.0E-04	G2
C10H9O8N1	10	9	8	1	-3.12	1.4E-02	2.7E-03	G2
C10H10O8N2	10	10	8	2	0.32	5.4E-03	1.8E-03	G2
C10H11O8N1	10	11	8	1	-3.12	9.3E-04	2.9E-04	G2
C10H12O8	10	12	8	0	-6.56	8.7E-04	5.7E-04	G2
C10H7O9N1	10	7	9	1	-4.84	2.0E-03	5.2E-04	G2
C10H8O9	10	8	9	0	-8.28	1.7E-03	6.2E-04	G2
C10H8O9N2	10	8	9	2	-1.4	5.2E-04	1.8E-04	G2
C10H10O9N2	10	10	9	2	-1.4	2.8E-03	9.6E-04	G2
C10H11O9N1	10	11	9	1	-4.84	8.5E-04	1.6E-04	G2
C10H7O10N1	10	7	10	1	-6.56	9.7E-04	3.0E-04	G2
C10H8O10	10	8	10	0	-10	3.3E-04	1.6E-04	G2

C10H9O10N1	10	9	10	1	-6.56	6.4E-03	1.8E-03	G2
C10H10O10N2	10	10	10	2	-3.12	6.0E-04	1.6E-04	G2
C10H11O10N1	10	11	10	1	-6.56	5.0E-04	1.2E-04	G2
C10H12O10	10	12	10	0	-10	3.5E-04	1.5E-04	G2
C10H8O11	10	8	11	0	-11.72	8.7E-05	1.7E-05	G2
C10H10O11	10	10	11	0	-11.72	2.0E-04	1.2E-04	G2
C10H10O11N2	10	10	11	2	-4.84	5.2E-04	1.3E-04	G2
C10H11O11N1	10	11	11	1	-8.28	3.9E-04	1.7E-04	G2
C10H12O11	10	12	11	0	-11.72	2.1E-04	9.8E-05	G2

Supplementary Table S7 Compound formulas measured by the PTR3, average yields and associated standard deviations (variation based on different runs) for toluene OH oxidation under no NO_x conditions. Compounds are distinguished between first- and second-generation products.

**Formula**	**C**	**H**	**O**	**N**	**LogC^***	**Yield**	**std**	**Generation**
C4H6O1	4	6	1	0	8.36	1.9E-02	1.2E-02	G1
C4H4O2	4	4	2	0	6.64	5.8E-03	1.6E-03	G1
C4H6O2	4	6	2	0	6.64	1.4E-02	5.9E-03	G1
C4H4O3	4	4	3	0	4.92	2.5E-03	7.2E-04	G1
C4H6O3	4	6	3	0	4.92	5.6E-03	1.6E-03	G1
C5H4O1	5	4	1	0	7.88	3.6E-03	8.8E-04	G1
C5H6O1	5	6	1	0	7.88	2.0E-02	5.4E-03	G1
C5H4O2	5	4	2	0	6.16	1.2E-02	5.9E-03	G1
C5H6O2	5	6	2	0	6.16	1.2E-01	6.8E-02	G1
C5H6O3	5	6	3	0	4.44	2.2E-02	7.2E-03	G1
C5H6O4	5	6	4	0	2.72	2.3E-03	1.1E-04	G1
C6H6O2	6	6	2	0	5.68	2.2E-02	1.0E-02	G1
C6H6O3	6	6	3	0	3.96	8.3E-03	3.1E-03	G1
C7H6O1	7	6	1	0	6.92	9.9E-02	4.2E-02	G1
C7H7O1	7	7	1	0	6.92	1.7E-03	4.8E-04	G1
C7H8O1	7	8	1	0	6.92	1.3E-01	6.6E-02	G1
C7H6O2	7	6	2	0	5.2	2.9E-02	1.1E-02	G1
C7H8O2	7	8	2	0	5.2	1.7E-02	5.5E-03	G1
C7H6O3	7	6	3	0	3.48	1.8E-02	4.3E-03	G1
C7H8O3	7	8	3	0	3.48	4.6E-02	1.3E-02	G1
C7H8O4	7	8	4	0	1.76	6.5E-02	1.7E-02	G1
C7H8O5	7	8	5	0	0.04	2.0E-03	5.4E-04	G1
C7H10O5	7	10	5	0	0.04	4.3E-03	1.9E-03	G1

Supplementary Table S8 Compound formulas measured by the PTR3, average yields and associated standard deviations (variation based on different runs) for toluene OH oxidation under high NO_x conditions (>0.2 ppbv). Compounds are distinguished between first- and second-generation products.

Formula	C	H	O	N	LogC^*	Yield	std	Generation
C4H6O1	4	6	1	0	8.36	1.9E-02	1.2E-02	G1
C4H4O2	4	4	2	0	6.64	1.9E-02	3.6E-03	G1
C4H6O2	4	6	2	0	6.64	1.4E-02	5.9E-03	G1
C4H4O3	4	4	3	0	4.92	5.1E-03	5.1E-03	G1
C4H6O3	4	6	3	0	4.92	5.6E-03	1.6E-03	G1
C5H4O1	5	4	1	0	7.88	4.2E-03	3.1E-04	G1
C5H6O1	5	6	1	0	7.88	4.8E-03	2.5E-04	G1
C5H4O2	5	4	2	0	6.16	1.2E-02	5.9E-03	G1
C5H6O2	5	6	2	0	6.16	1.2E-01	6.8E-02	G1
C5H6O3	5	6	3	0	4.44	2.2E-02	7.2E-03	G1
C5H6O4	5	6	4	0	2.72	2.3E-03	1.1E-04	G1
C6H8O1	6	8	1	0	7.4	1.0E-03	1.1E-04	G1
C6H6O2	6	6	2	0	5.68	2.2E-02	1.0E-02	G1
C6H8O2	6	8	2	0	5.68	4.5E-03	3.6E-04	G1
C6H6O3	6	6	3	0	3.96	8.3E-03	3.1E-03	G1
C7H6O1	7	6	1	0	6.92	9.9E-02	4.2E-02	G1
C7H7O1	7	7	1	0	6.92	1.2E-02	2.0E-03	G1
C7H8O1	7	8	1	0	6.92	1.3E-01	6.6E-02	G1
C7H10O1	7	10	1	0	6.92	5.2E-04	3.5E-05	G1
C7H6O2	7	6	2	0	5.2	2.9E-02	1.1E-02	G1
C7H8O2	7	8	2	0	5.2	1.7E-02	5.5E-03	G1
C7H10O2	7	10	2	0	5.2	8.9E-04	9.7E-05	G1
C7H6O3	7	6	3	0	3.48	1.3E-02	1.2E-03	G1
C7H7O3N1	7	7	3	1	6.92	9.9E-02	1.7E-02	G1
C7H8O3	7	8	3	0	3.48	1.4E-02	1.4E-03	G1
C7H10O3	7	10	3	0	3.48	1.2E-03	2.1E-04	G1
C7H8O4	7	8	4	0	1.76	2.3E-02	1.3E-03	G1
C7H9O4N1	7	9	4	1	5.2	6.1E-03	8.5E-04	G1
C7H7O5N1	7	7	5	1	3.48	4.5E-03	2.7E-04	G1
C7H8O5	7	8	5	0	0.04	1.2E-03	1.0E-04	G1
C7H9O5N1	7	9	5	1	3.48	7.0E-04	1.5E-04	G1
C7H10O5	7	10	5	0	0.04	4.3E-03	1.9E-03	G1
C7H9O6N1	7	9	6	1	1.76	1.9E-03	1.2E-04	G1
C7H9O7N1	7	9	7	1	0.04	2.4E-04	4.2E-05	G1
C6H5O3N1	6	5	3	1	7.4	4.5E-02	2.0E-02	G2
C5H10O2	5	10	2	0	6.16	3.4E-04	3.3E-04	G2

Supplementary Table S9 Compound formulas measured by the PTR3, average yields and associated standard deviations (variation based on different runs) for trimethylbenzene OH oxidation under no NO_x conditions. Compounds are distinguished between first- and second-generation products.

Formula	C	H	O	N	LogC^*	Yield	std	Generation
C4H6O1	4	6	1	0	8.36	2.7E-03	8.8E-04	G1
C4H8O1	4	8	1	0	8.36	8.7E-04	4.2E-04	G1
C4H8O1N1	4	8	1	1	11.8	1.2E-03	6.3E-04	G1
C4H4O2	4	4	2	0	6.64	6.8E-04	2.5E-04	G1
C4H6O2	4	6	2	0	6.64	9.2E-03	2.0E-03	G1
C4H8O2	4	8	2	0	6.64	1.2E-03	6.9E-04	G1
C4H4O3	4	4	3	0	4.92	3.4E-04	1.3E-04	G1
C4H6O3	4	6	3	0	4.92	9.6E-03	3.6E-03	G1
C4H6O4	4	6	4	0	3.2	2.1E-04	5.9E-05	G1
C5H8O1	5	8	1	0	7.88	8.1E-03	2.0E-03	G1
C5H4O2	5	4	2	0	6.16	8.1E-04	2.6E-04	G1
C5H6O2	5	6	2	0	6.16	3.6E-03	8.2E-04	G1
C5H8O2	5	8	2	0	6.16	1.1E-02	3.5E-03	G1
C5H10O2	5	10	2	0	6.16	7.5E-04	3.4E-04	G1
C5H6O3	5	6	3	0	4.44	1.3E-03	3.2E-04	G1
C5H8O3	5	8	3	0	4.44	6.5E-03	1.8E-03	G1
C5H6O4	5	6	4	0	2.72	5.2E-04	1.6E-04	G1
C6H6O1	6	6	1	0	7.4	1.8E-03	4.4E-04	G1
C6H8O1	6	8	1	0	7.4	2.1E-02	6.3E-03	G1
C6H6O2	6	6	2	0	5.68	2.6E-03	5.5E-04	G1
C6H8O2	6	8	2	0	5.68	1.6E-01	3.5E-02	G1
C6H10O2	6	10	2	0	5.68	3.7E-03	1.6E-03	G1
C6H6O3	6	6	3	0	3.96	8.9E-04	2.1E-04	G1
C6H10O3	6	10	3	0	3.96	6.0E-03	1.2E-03	G1
C6H6O4	6	6	4	0	2.24	2.2E-04	8.2E-05	G1
C6H10O4	6	10	4	0	2.24	1.5E-03	4.4E-04	G1
C6H10O5	6	10	5	0	0.52	3.3E-04	1.3E-04	G1
C7H8O1	7	8	1	0	6.92	8.0E-03	2.3E-03	G1
C7H10O1	7	10	1	0	6.92	8.8E-03	2.3E-03	G1
C7H8O2	7	8	2	0	5.2	2.7E-02	7.0E-03	G1
C7H9O2	7	9	2	0	5.2	1.3E-03	3.3E-04	G1
C7H10O2	7	10	2	0	5.2	3.3E-02	7.5E-03	G1
C7H6O3	7	6	3	0	3.48	9.8E-04	2.6E-04	G1
C7H8O3	7	8	3	0	3.48	2.4E-02	5.9E-03	G1
C7H10O3	7	10	3	0	3.48	5.0E-03	1.2E-03	G1
C7H12O3	7	12	3	0	3.48	5.5E-04	3.5E-04	G1
C7H8O4	7	8	4	0	1.76	2.4E-03	5.1E-04	G1
C8H10O1	8	10	1	0	6.44	6.4E-03	1.4E-03	G1

C8H12O1	8	12	1	0	6.44	1.6E-03	6.4E-04	G1
C8H8O2	8	8	2	0	4.72	1.2E-03	3.1E-04	G1
C8H10O2	8	10	2	0	4.72	2.5E-02	5.5E-03	G1
C8H12O2	8	12	2	0	4.72	6.3E-03	2.8E-03	G1
C8H8O3	8	8	3	0	3	8.1E-04	2.3E-04	G1
C8H10O3	8	10	3	0	3	1.7E-02	4.7E-03	G1
C8H10O4	8	10	4	0	1.28	1.9E-03	7.0E-04	G1
C8H12O4	8	12	4	0	1.28	2.4E-03	6.8E-04	G1
C8H10O5	8	10	5	0	-0.44	4.1E-04	1.4E-04	G1
C9H8O1	9	8	1	0	5.96	1.4E-03	3.4E-04	G1
C9H10O1	9	10	1	0	5.96	5.9E-02	1.3E-02	G1
C9H11O1	9	11	1	0	5.96	2.6E-03	6.4E-04	G1
C9H12O1	9	12	1	0	5.96	1.0E-02	3.5E-03	G1
C9H8O2	9	8	2	0	4.24	3.9E-03	8.4E-04	G1
C9H10O2	9	10	2	0	4.24	1.4E-02	3.4E-03	G1
C9H12O2	9	12	2	0	4.24	4.2E-03	1.0E-03	G1
C9H8O3	9	8	3	0	2.52	4.8E-04	3.2E-04	G1
C9H10O3	9	10	3	0	2.52	1.2E-02	3.0E-03	G1
C9H12O3	9	12	3	0	2.52	8.1E-03	1.9E-03	G1
C9H8O4	9	8	4	0	0.8	7.8E-04	2.7E-04	G1
C9H10O4	9	10	4	0	0.8	2.7E-03	3.8E-04	G1
C9H12O4	9	12	4	0	0.8	2.6E-02	6.9E-03	G1
C9H14O4	9	14	4	0	0.8	7.9E-04	2.4E-04	G1
C9H10O5	9	10	5	0	-0.92	8.1E-04	2.0E-04	G1
C9H12O5	9	12	5	0	-0.92	1.9E-03	6.0E-04	G1
C9H14O5	9	14	5	0	-0.92	2.9E-03	8.8E-04	G1
C9H12O6	9	12	6	0	-2.64	6.6E-04	2.1E-04	G1
C9H14O6	9	14	6	0	-2.64	1.2E-04	3.2E-05	G1

Supplementary Table S10 Compound formulas measured by the PTR3, average yields and associated standard deviations (variation based on different runs) for trimethylbenzene OH oxidation under high NO_x conditions (>0.2 ppbv). Compounds are distinguished between first- and second-generation products.

**Formula**	**C**	**H**	**O**	**N**	**Log*C****	**Yield**	**std**	**Generation**
C4H6O1	4	6	1	0	8.36	5.4E-03	8.8E-04	G1
C4H8O1	4	8	1	0	8.36	3.3E-04	1.2E-04	G1
C4H8O1N1	4	8	1	1	11.8	1.2E-03	6.3E-04	G1
C4H4O2	4	4	2	0	6.64	1.1E-03	3.0E-04	G1
C4H6O2	4	6	2	0	6.64	2.6E-02	6.1E-03	G1
C4H8O2	4	8	2	0	6.64	1.2E-03	6.9E-04	G1
C4H4O3	4	4	3	0	4.92	7.8E-04	1.6E-04	G1
C4H6O3	4	6	3	0	4.92	9.6E-03	3.6E-03	G1
C4H6O4	4	6	4	0	3.2	2.1E-04	5.9E-05	G1
C5H8O1	5	8	1	0	7.88	8.1E-03	2.0E-03	G1
C5H4O2	5	4	2	0	6.16	2.5E-03	4.9E-04	G1
C5H5O2	5	5	2	0	6.16	1.6E-04	6.5E-05	G1
C5H6O2	5	6	2	0	6.16	1.1E-02	1.8E-03	G1
C5H8O2	5	8	2	0	6.16	1.1E-02	3.5E-03	G1
C5H10O2	5	10	2	0	6.16	3.5E-04	8.1E-05	G1
C5H6O3	5	6	3	0	4.44	4.5E-03	8.4E-04	G1
C5H8O3	5	8	3	0	4.44	8.3E-03	2.2E-03	G1
C5H6O4	5	6	4	0	2.72	5.2E-04	1.6E-04	G1
C6H6O1	6	6	1	0	7.4	3.9E-03	7.6E-04	G1
C6H7O1	6	7	1	0	7.4	3.2E-04	7.1E-05	G1
C6H8O1	6	8	1	0	7.4	5.5E-03	9.7E-04	G1
C6H6O2	6	6	2	0	5.68	4.8E-03	1.0E-03	G1
C6H7O2	6	7	2	0	5.68	4.9E-04	1.5E-04	G1
C6H8O2	6	8	2	0	5.68	3.4E-01	6.5E-02	G1
C6H10O2	6	10	2	0	5.68	1.8E-03	4.7E-04	G1
C6H6O3	6	6	3	0	3.96	8.9E-04	2.1E-04	G1
C6H10O3	6	10	3	0	3.96	6.0E-03	1.2E-03	G1
C6H6O4	6	6	4	0	2.24	2.2E-04	8.2E-05	G1
C6H10O4	6	10	4	0	2.24	2.9E-03	8.0E-04	G1
C6H10O5	6	10	5	0	0.52	5.3E-04	2.0E-04	G1
C7H8O1	7	8	1	0	6.92	1.4E-02	3.0E-03	G1
C7H10O1	7	10	1	0	6.92	6.2E-03	1.4E-03	G1
C7H8O2	7	8	2	0	5.2	1.8E-02	3.4E-03	G1
C7H9O2	7	9	2	0	5.2	1.3E-03	3.3E-04	G1
C7H10O2	7	10	2	0	5.2	5.8E-02	1.1E-02	G1
C7H6O3	7	6	3	0	3.48	6.5E-04	7.5E-05	G1
C7H7O3N1	7	7	3	1	6.92	2.7E-03	8.8E-04	G1
C7H8O3	7	8	3	0	3.48	1.1E-02	1.8E-03	G1

C7H10O3	7	10	3	0	3.48	2.7E-03	4.4E-04	G1
C7H8O4	7	8	4	0	1.76	2.4E-03	5.1E-04	G1
C7H9O5N1	7	9	5	1	3.48	2.6E-04	5.4E-05	G1
C8H10O1	8	10	1	0	6.44	6.4E-03	1.1E-03	G1
C8H12O1	8	12	1	0	6.44	7.4E-04	1.8E-04	G1
C8H8O2	8	8	2	0	4.72	1.2E-03	3.1E-04	G1
C8H10O2	8	10	2	0	4.72	1.3E-02	2.4E-03	G1
C8H12O2	8	12	2	0	4.72	1.6E-03	3.6E-04	G1
C8H8O3	8	8	3	0	3	8.1E-04	2.3E-04	G1
C8H10O3	8	10	3	0	3	1.4E-02	5.3E-03	G1
C8H10O4	8	10	4	0	1.28	1.9E-03	7.0E-04	G1
C8H12O4	8	12	4	0	1.28	1.3E-03	2.9E-04	G1
C8H10O5	8	10	5	0	-0.44	4.0E-04	9.5E-05	G1
C9H8O1	9	8	1	0	5.96	5.6E-03	1.7E-03	G1
C9H10O1	9	10	1	0	5.96	5.9E-02	1.3E-02	G1
C9H11O1	9	11	1	0	5.96	1.0E-02	1.6E-03	G1
C9H12O1	9	12	1	0	5.96	1.0E-02	3.5E-03	G1
C9H8O2	9	8	2	0	4.24	9.8E-03	1.3E-03	G1
C9H9O2N1	9	9	2	1	7.68	2.3E-04	7.7E-05	G1
C9H10O2	9	10	2	0	4.24	1.4E-02	3.1E-03	G1
C9H12O2	9	12	2	0	4.24	4.2E-03	1.0E-03	G1
C9H9O3N1	9	9	3	1	5.96	4.2E-05	7.9E-06	G1
C9H10O3	9	10	3	0	2.52	1.2E-02	3.0E-03	G1
C9H11O3N1	9	11	3	1	5.96	9.4E-03	1.4E-03	G1
C9H12O3	9	12	3	0	2.52	8.9E-03	1.7E-03	G1
C9H8O4	9	8	4	0	0.8	7.9E-04	1.9E-04	G1
C9H9O4N1	9	9	4	1	4.24	2.3E-04	6.7E-05	G1
C9H10O4	9	10	4	0	0.8	1.3E-03	2.3E-04	G1
C9H12O4	9	12	4	0	0.8	1.4E-02	2.4E-03	G1
C9H14O4	9	14	4	0	0.8	5.4E-04	1.1E-04	G1
C9H10O5	9	10	5	0	-0.92	4.7E-04	9.6E-05	G1
C9H11O5N1	9	11	5	1	2.52	1.1E-02	2.6E-03	G1
C9H12O5	9	12	5	0	-0.92	1.9E-03	6.0E-04	G1
C9H13O5N1	9	13	5	1	2.52	7.5E-04	9.1E-05	G1
C9H14O5	9	14	5	0	-0.92	1.6E-03	2.8E-04	G1
C9H11O6N1	9	11	6	1	0.8	1.2E-03	3.9E-04	G1
C9H12O6	9	12	6	0	-2.64	6.6E-04	2.1E-04	G1
C9H13O6N1	9	13	6	1	0.8	4.3E-03	9.4E-04	G1
C9H14O6	9	14	6	0	-2.64	2.8E-04	1.7E-04	G1
C9H11O7N1	9	11	7	1	-0.92	3.5E-04	9.7E-05	G1
C9H13O7N1	9	13	7	1	-0.92	6.6E-04	1.8E-04	G1
C9H8O3	9	8	3	0	2.52	3.2E-02	3.5E-02	G2
C9H9O5N1	9	9	5	1	2.52	1.4E-03	1.6E-03	G2
C7H12O3	7	12	3	0	3.48	6.8E-04	1.7E-03	G2

SupplementaryTable S11 Compound formulas measured by the PTR3, average yields and associated standard deviations (variation based on different runs) for naphthalene OH oxidation under no NO_x conditions. Compounds are distinguished between first- and second-generation products.

**Formula**	**C**	**H**	**O**	**N**	**Log*C****	**Yield**	**std**	**Generation**
C5H8O2	5	8	2	0	6.16	3.3E-02	2.5E-02	G1
C6H10O2	6	10	2	0	5.68	5.2E-02	2.6E-02	G1
C6H10O4	6	10	4	0	2.24	4.8E-03	2.6E-03	G1
C7H6O1	7	6	1	0	6.92	1.8E-03	1.8E-03	G1
C7H6O2	7	6	2	0	5.2	8.0E-03	4.0E-03	G1
C7H6O3	7	6	3	0	3.48	3.8E-03	2.6E-03	G1
C8H10O1	8	10	1	0	6.44	4.0E-04	2.2E-04	G1
C8H12O1	8	12	1	0	6.44	1.6E-03	1.9E-03	G1
C8H8O2	8	8	2	0	4.72	7.6E-04	4.3E-04	G1
C8H8O3	8	8	3	0	3	6.7E-04	4.6E-04	G1
C9H8O1	9	8	1	0	5.96	3.1E-03	4.3E-04	G1
C9H10O1	9	10	1	0	5.96	1.6E-03	6.8E-04	G1
C9H8O2	9	8	2	0	4.24	3.0E-03	1.1E-03	G1
C9H10O2	9	10	2	0	4.24	4.3E-04	6.4E-04	G1
C9H10O3	9	10	3	0	2.52	1.3E-03	6.3E-04	G1
C10H8O1	10	8	1	0	5.48	5.3E-01	1.4E-01	G1
C10H8O2	10	8	2	0	3.76	9.7E-02	7.7E-03	G1
C10H10O2	10	10	2	0	3.76	4.4E-03	4.2E-04	G1
C10H8O3	10	8	3	0	2.04	6.0E-02	1.1E-02	G1
C10H10O3	10	10	3	0	2.04	1.3E-03	2.6E-04	G1
C10H10O4	10	10	4	0	0.32	1.7E-03	2.2E-04	G1
C9H8O3	9	8	3	0	2.52	3.3E-02	2.3E-02	G2
C9H8O4	9	8	4	0	0.8	2.0E-02	1.2E-02	G2
C9H8O5	9	8	5	0	-0.92	4.6E-03	2.9E-03	G2
C10H8O4	10	8	4	0	0.32	3.7E-01	1.5E-01	G2
C10H8O5	10	8	5	0	-1.4	4.2E-02	1.8E-02	G2
C10H10O5	10	10	5	0	-1.4	4.6E-02	1.4E-02	G2

Supplementary Table S12 Compound formulas measured by the PTR3, average yields and associated standard deviations (variation based on different runs) for naphthalene OH oxidation under high NO_x conditions (>0.2 ppbv). Compounds are distinguished between first- and second-generation products.

Formula	C	H	O	N	LogC^*	Yield	std	Generation
C5H8O2	5	8	2	0	6.16	3.6E-03	3.4E-03	G1
C6H10O2	6	10	2	0	5.68	8.6E-03	3.8E-03	G1
C6H10O4	6	10	4	0	2.24	4.8E-03	2.6E-03	G1
C6H10O5	6	10	5	0	0.52	1.6E-03	2.8E-04	G1
C7H6O1	7	6	1	0	6.92	5.8E-03	1.5E-03	G1
C7H6O2	7	6	2	0	5.2	1.1E-02	4.1E-03	G1
C7H6O3	7	6	3	0	3.48	3.8E-03	2.6E-03	G1
C8H10O1	8	10	1	0	6.44	4.0E-04	2.2E-04	G1
C8H12O1	8	12	1	0	6.44	1.6E-03	1.9E-03	G1
C8H8O2	8	8	2	0	4.72	2.3E-03	2.8E-04	G1
C8H8O3	8	8	3	0	3	1.5E-03	3.2E-04	G1
C9H8O1	9	8	1	0	5.96	2.0E-02	4.7E-03	G1
C9H10O1	9	10	1	0	5.96	5.7E-03	1.3E-03	G1
C9H8O2	9	8	2	0	4.24	5.9E-03	1.4E-03	G1
C9H9O2N1	9	9	2	1	7.68	4.3E-04	6.8E-05	G1
C9H10O2	9	10	2	0	4.24	1.2E-03	2.0E-04	G1
C9H10O3	9	10	3	0	2.52	1.3E-03	6.3E-04	G1
C10H8O1	10	8	1	0	5.48	5.3E-01	1.4E-01	G1
C10H8O2	10	8	2	0	3.76	4.1E-01	6.8E-02	G1
C10H9O2N1	10	9	2	1	7.2	7.5E-04	1.7E-04	G1
C10H10O2	10	10	2	0	3.76	6.3E-03	6.6E-04	G1
C10H8O3	10	8	3	0	2.04	6.0E-02	1.1E-02	G1
C10H9O3N1	10	9	3	1	5.48	1.5E-03	4.5E-04	G1
C10H10O3	10	10	3	0	2.04	2.3E-03	4.8E-04	G1
C10H9O4N1	10	9	4	1	3.76	1.6E-03	2.5E-04	G1
C10H10O4	10	10	4	0	0.32	2.6E-03	4.4E-04	G1
C10H9O6N1	10	9	6	1	0.32	1.4E-03	3.5E-04	G1
C10H9O7N1	10	9	7	1	-1.4	6.3E-04	1.1E-04	G1
C9H8O3	9	8	3	0	2.52	4.3E-02	2.2E-02	G2
C9H8O4	9	8	4	0	0.8	2.8E-02	1.3E-02	G2
C9H8O5	9	8	5	0	-0.92	6.3E-03	4.7E-03	G2
C10H8O4	10	8	4	0	0.32	4.1E-01	1.8E-01	G2
C10H8O5	10	8	5	0	-1.4	4.4E-02	2.7E-02	G2
C10H10O5	10	10	5	0	-1.4	4.1E-02	1.5E-02	G2

Supplementary Table S13 Experimental VOC, OH, NO concentrations and apparent HOMs yields. TOL:Toluene experiments, NAPH: Naphthalene experiments, TMB: Trimethylbenzene experiments, N.I.: Not injected.

run ID	VOCs	VOCs (pptv)	OH (molec cm^{-3})	NO (ppbv)	Apparent HOMs yield	$HOMs_{Ncontain}$ over $HOMs_{all}$
1828.02	TOL	36174	1.11E+06	N.I.	0.02	/
1828.06	TOL	37612	1.45E+06	N.I.	0.021	/
1831.02	TOL	34451	1.06E+06	N.I.	0.021	/
1831.03	TOL	37376	1.77E+06	N.I.	0.023	/
1831.04	TOL	37457	1.38E+06	N.I.	0.019	/
1831.06	TOL	37635	1.59E+06	N.I.	0.019	/
1831.09	TOL	37615	1.63E+06	N.I.	0.022	/
1831.12	TOL	37626	1.65E+06	N.I.	0.024	/
1831.11	TOL	37460	2.15E+06	N.I.	0.025	/
1832.01	TOL	22570	1.08E+06	N.I.	0.017	/
1832.06	TOL	18975	2.80E+06	N.I.	0.022	/
1839.02	TOL	37539	1.93E+06	0.1	0.01	0.41
1839.03	TOL	37186	2.66E+06	0.078	0.014	0.32
1839.04	TOL	36838	3.35E+06	0.066	0.015	0.27
1839.12	TOL	37332	3.18E+06	0.07	0.017	0.27
1839.13	TOL	36539	3.46E+06	0.057	0.02	0.22
1840.01	TOL	37854	2.83E+06	0.46	0.009	0.65
1840.02	TOL	36111	5.02E+06	0.373	0.012	0.61
1840.03	TOL	34967	5.97E+06	0.306	0.017	0.56
1840.05	TOL	35087	6.60E+06	0.324	0.015	0.56
1840.07	TOL	36559	4.29E+06	0.202	0.013	0.54
1840.08	TOL	35677	5.00E+06	0.185	0.015	0.5
1840.09	TOL	34400	6.03E+06	0.162	0.019	0.45
1829.06	NAPH	96	7.84E+06	N.I.	0.042	/
1829.07	NAPH	90	7.30E+06	N.I.	0.057	/
1829.14	NAPH	130	3.79E+06	N.I.	0.038	/
1829.15	NAPH	106	5.64E+06	N.I.	0.045	/
1829.21	NAPH	1444	1.28E+06	N.I.	0.021	/
1829.22	NAPH	1224	2.43E+06	N.I.	0.026	/
1829.25	NAPH	1111	2.86E+06	N.I.	0.028	/
1829.26	NAPH	1110	4.05E+06	N.I.	0.038	/
1829.29	NAPH	2597	3.62E+06	N.I.	0.031	/
1834.02	NAPH	1815	8.34E+05	0.766	0.009	0.46
1834.03	NAPH	1792	1.78E+06	0.764	0.008	0.46
1834.04	NAPH	1655	2.07E+06	0.734	0.009	0.46
1834.05	NAPH	1497	2.28E+06	0.715	0.011	0.46
1834.06	NAPH	1368	3.02E+06	0.702	0.011	0.46

1835.02	NAPH	1878	2.80E+06	0.07	0.011	0.33
1835.03	NAPH	1461	4.68E+06	0.058	0.018	0.26
1835.05	NAPH	1790	4.11E+06	0.065	0.014	0.28
1835.06	NAPH	1431	4.77E+06	0.055	0.02	0.25
1836.02	NAPH	1678	3.36E+06	0.188	0.01	0.41
1836.03	NAPH	1236	5.83E+06	0.156	0.016	0.36
1836.04	NAPH	968	5.52E+06	0.13	0.028	0.31
1836.07	NAPH	971	6.52E+06	0.127	0.031	0.29
1838.02	NAPH	4517	1.25E+06	0.265	0.009	0.42
1838.03	NAPH	3700	2.53E+06	0.228	0.013	0.4
1838.04	NAPH	3284	3.51E+06	0.195	0.017	0.36
1838.06	NAPH	3418	3.80E+06	0.2	0.019	0.35
1838.08	NAPH	3063	3.88E+06	0.178	0.023	0.33
1838.1	NAPH	4211	1.46E+06	0.467	0.009	0.44
1838.11	NAPH	3261	2.76E+06	0.408	0.013	0.43
1838.14	NAPH	2679	3.93E+06	0.337	0.019	0.39
1838.15	NAPH	2668	4.04E+06	0.336	0.024	0.38
1838.16	NAPH	3648	4.61E+06	0.318	0.016	0.37
1830.02	TMB	3183	1.09E+06	N.I.	0.02	/
1830.03	TMB	2735	1.91E+06	N.I.	0.022	/
1830.04	TMB	2768	3.02E+06	N.I.	0.028	/
1830.05	TMB	2728	2.21E+06	N.I.	0.023	/
1830.07	TMB	2832	2.45E+06	N.I.	0.023	/
1830.08	TMB	2484	2.99E+06	N.I.	0.025	/
1830.11	TMB	2553	3.03E+06	N.I.	0.032	/
1841.01	TMB	6785	1.43E+06	0.16	0.006	0.38
1841.02	TMB	6267	2.48E+06	0.131	0.009	0.34
1841.03	TMB	5632	3.01E+06	0.105	0.011	0.3
1841.05	TMB	5773	3.28E+06	0.105	0.013	0.29
1841.09	TMB	6548	2.05E+06	0.156	0.009	0.34
1841.11	TMB	5692	3.36E+06	0.118	0.012	0.3
1841.12	TMB	4918	4.69E+06	0.095	0.014	0.24
1841.14	TMB	4519	3.97E+06	0.084	0.021	0.23
1842.01	TMB	7578	8.74E+05	0.608	0.006	0.5
1842.03	TMB	6740	1.97E+06	0.462	0.007	0.52
1842.04	TMB	6049	2.83E+06	0.408	0.008	0.51
1842.07	TMB	5811	3.64E+06	0.351	0.008	0.5
1842.1	TMB	7437	1.29E+06	0.79	0.005	0.52
1842.11	TMB	6478	2.08E+06	0.801	0.006	0.55
1842.12	TMB	5401	2.75E+06	0.76	0.007	0.55
1842.14	TMB	5192	3.79E+06	0.706	0.008	0.54

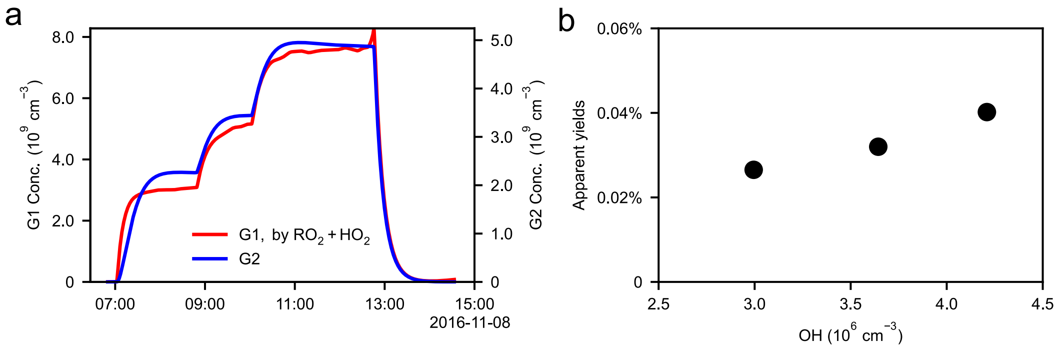

Supplementary Fig. S1. (a) Modelled first-generation products produced due to a peroxy radical terminated by HO_2 radical (red line, left axis) and second-generation products (blue line, right axis). (b) Apparent yield of first-generation products produced due to a peroxy radical terminated by HO_2 radical, showing an increase with OH concentration, due to the covariance between OH and HO_2. The figure shows that the increase of a compound concentration and yield with OH is not sufficient to distinguish between first- and second-generation products. Therefore, we used the appearance time (Extended Data Figure 3) as an additional criterion distinguishing between first- and second-generation products.

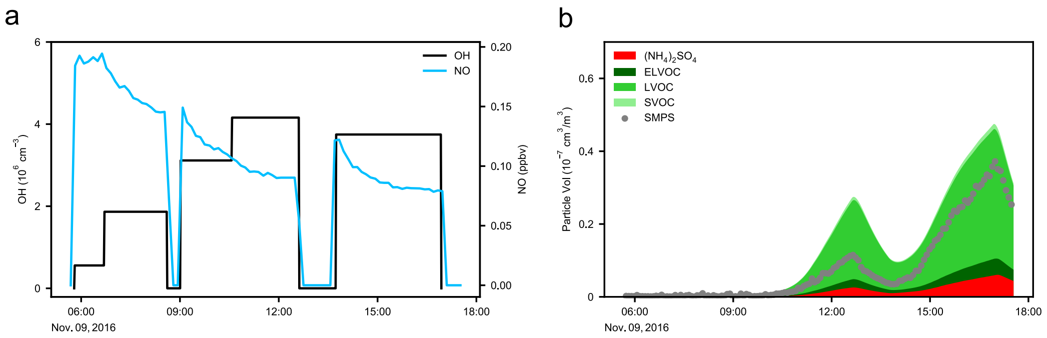

Supplementary Fig. S2. Secondary aerosol formed in an 1,2,4-trimethylbenzene oxidation experiment. **(a)** Trimethylbenzene oxidation at increasing OH concentration (black solid line, left axis) and relatively constant NO level (sky blue solid line, right axis). **(b)** Volume of SOA (above 6 nm) formed from trimethylbenzene oxidation. The red, dark green, medium green and light green areas are the modelled volume concentrations of sulfate, extremely low-volatility compounds (ELVOC), low-volatility compounds (LVOC) and semi-volatile compounds (SVOC), respectively, while the solid circles represent the volume based on the SMPS measurements.

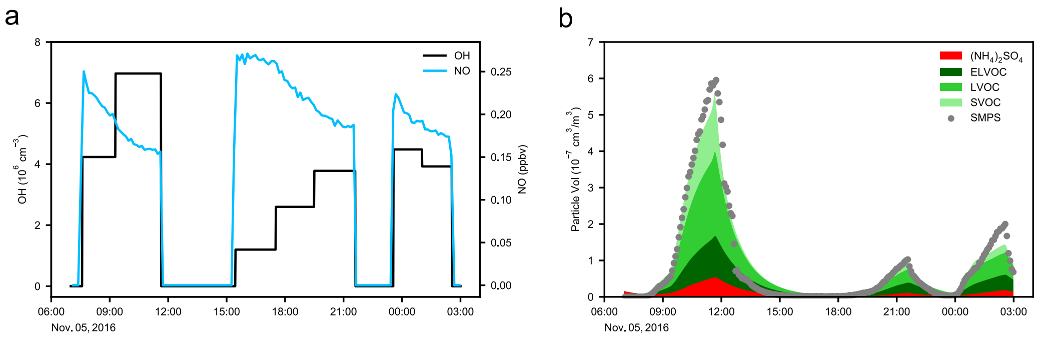

Supplementary Fig. S3. Secondary aerosol formed in a naphthalene oxidation experiment. **(a)** Naphthalene oxidation at increasing OH concentration (left scale: black solid line) and relatively constant NO level (right: sky blue solid line). **(b)** Volume of SOA (above 6 nm) formed from naphthalene oxidation. The red, dark green, medium green and light green areas are the modelled volume concentrations of sulfate, extremely low-volatility compounds (ELVOC), low-volatility compounds (LVOC) and semi-volatile compounds (SVOC), respectively, while the solid circles represent the volume based on the SMPS measurements.

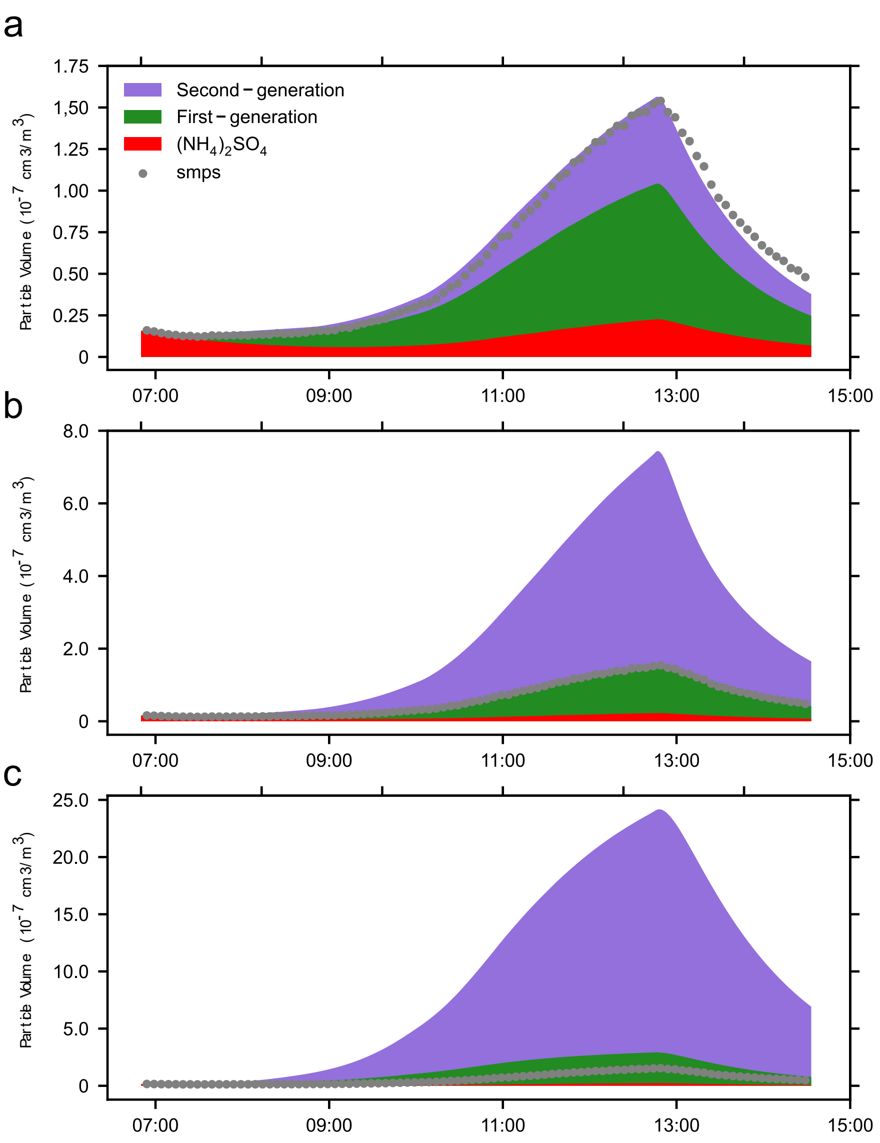

Supplementary Fig. S4. Modelled volume concentration of secondary aerosol formed from sulfuric acid and toluene oxidation products. The red, green and purple areas are the modelled volume concentrations of sulfate, first-generation compounds and second-generation compounds, respectively, while the solid circles represent the volume based on the SMPS measurements. In (**a**), gas phase oxidation product concentrations are modelled based on their yields and respective production and loss rates. To remove the wall influence and extend to the ambient atmosphere, in (**b**) first-generation oxidation products that produce $G2_{ww}$ are excluded from wall loss. Finally, in **(c)**, both first- and second-generation products are excluded from wall loss. Removing wall loss entirely results in a ten-fold increase in particle volume, almost entirely from second-generation species.

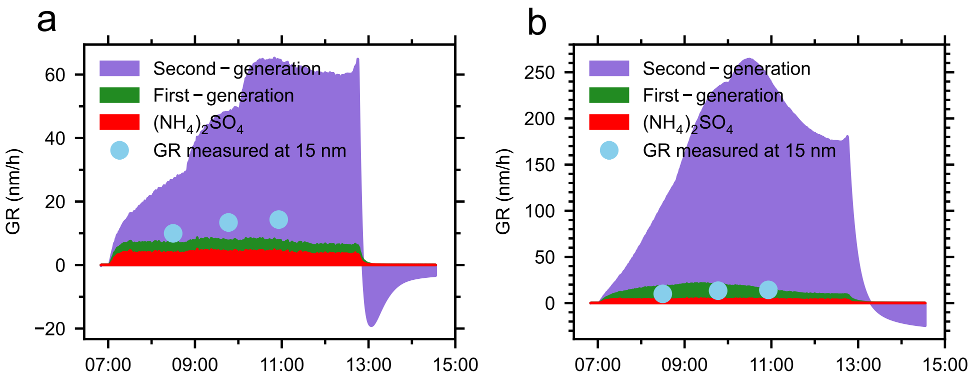

Supplementary Fig. S5. Growth rate contributions. (a) Growth by $(NH_4)_2SO_4$ (red), first- (green) and second-generation (purple) toluene oxidation products if first-generation oxidation products that produce $G2_{ww}$ are not lost to the chamber wall. **(b)** Growth by $(NH_4)_2SO_4$ (red), first- (green) and second-generation (purple) toluene oxidation products if first- and second- generation oxidation products are not lost to the wall. Sky blue circles represent measured growth rates.

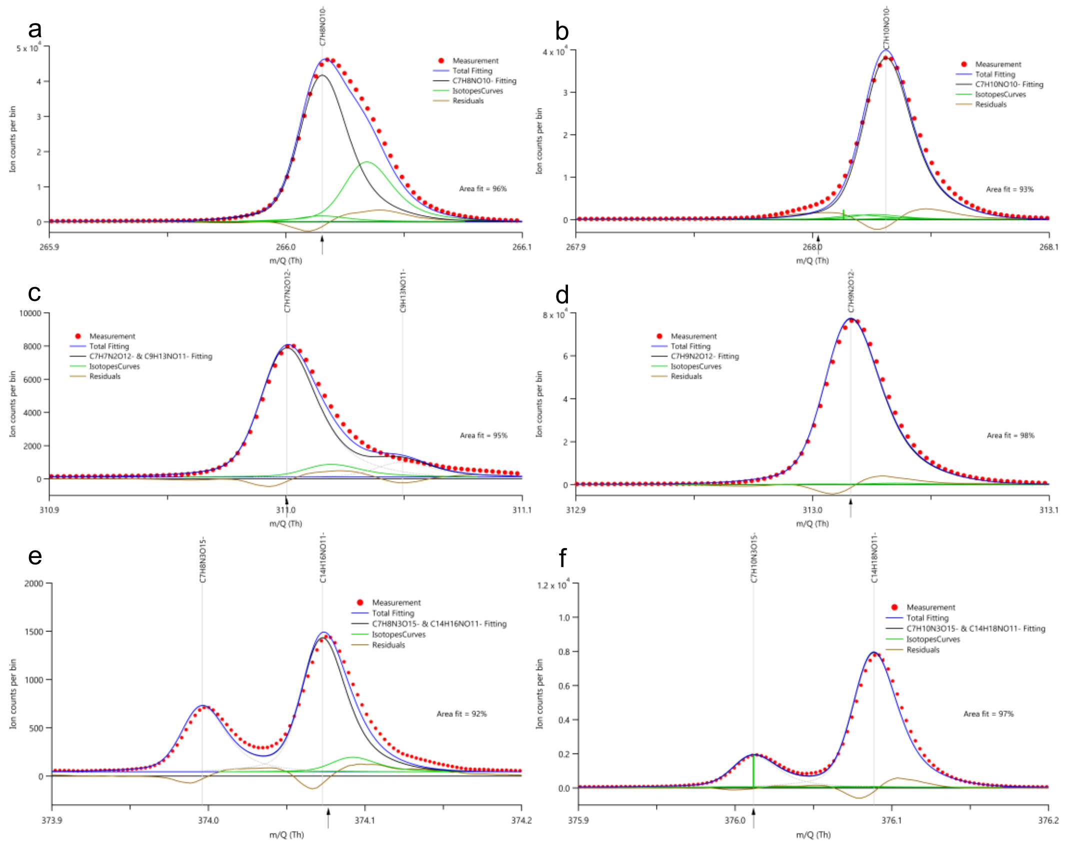

Supplementary Fig. S6. Peaks fitted at *m/z* 266, 268, 311, 313, 374 and 376 from a toluene experiment from 2016-11-08. Red dots present measured spectra, blue lines, black lines and green lines are sum of fitted peaks, fitted peaks and isotopic peaks, respectively. The olive color lines show the fitting residuals. These masses present typical first- (C7H8O7, C7H9O7NO2) and second-generation monomers (C7H10O7, C7H7O7NO2) and dimers (C14H16O7, C14H10O7) in the toluene experiment.

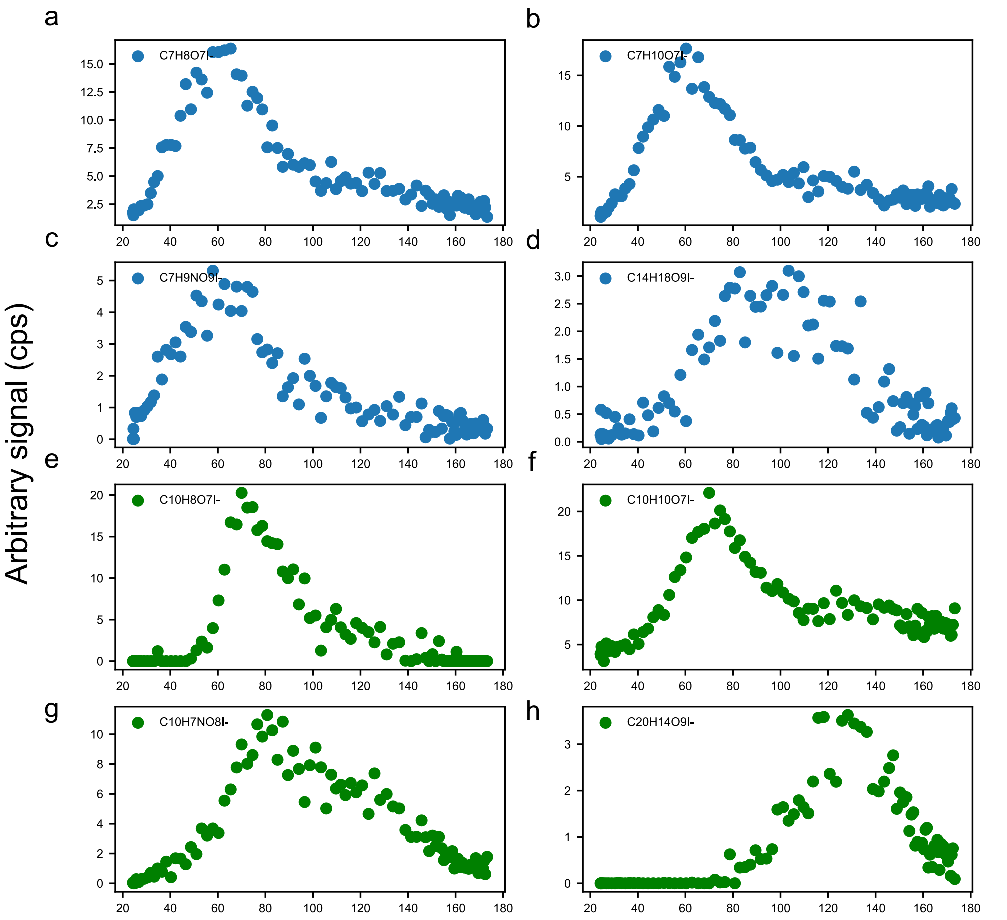

Supplementary Fig. S7. Figaero thermograms of (a) C7H8O7, (b) C7H10O7, (c) C7H9NO9, (d) C14H18O9 from a toluene oxidation experiment and (e) C10H8O7, (f) C10H10O7, (g) C10H7NO8 and (h) C20H14O9 from a naphthalene oxidation experiment.

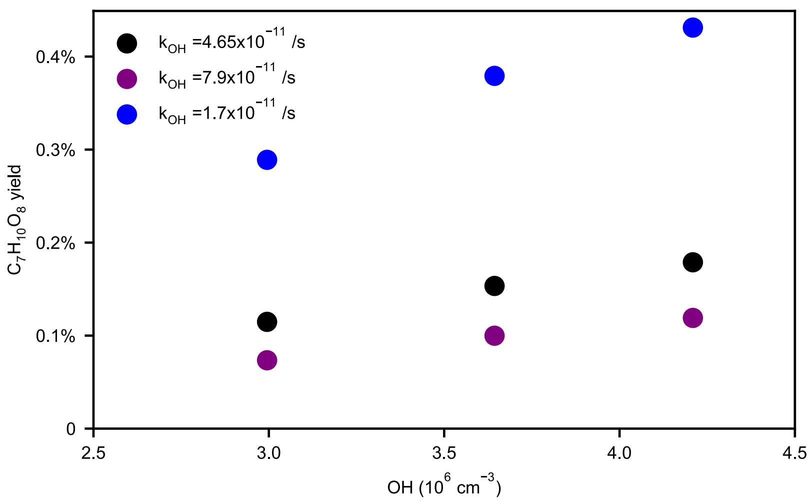

Supplementary Fig. S8. Yields of $C_7H_{10}O_8$, a G2 formed from a $G1_W$ through HO_2 or RO_2 pathway, vs. OH concentrations using 3 reaction-rate coefficients: 1.7 × 10^{-11} molecule^{-1} cm^3 s^{-1} (blue), 4.7 × 10^{-11} molecule^{-1} cm^3 s^{-1} (black), and 7.9 × 10^{-11} molecule^{-1} cm^3 s^{-1} (purple).

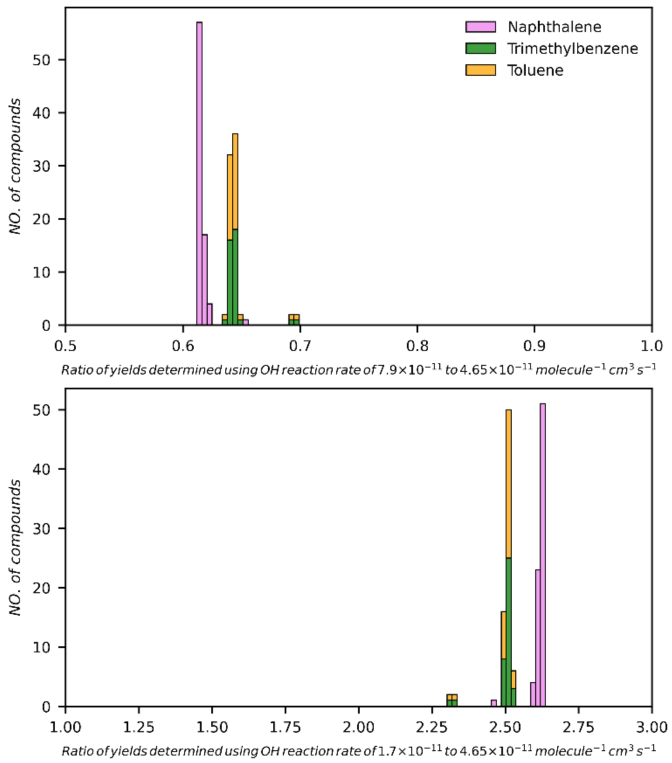

Supplementary Fig. S9. Changes of the yields as a function of OH rate constants.
